# Microwave-assisted enzymatic synthesis of geraniol esters in solvent-free systems: optimization of the reaction parameters, purification and characterization of the products, and biocatalyst reuse

**DOI:** 10.1007/s11030-023-10682-y

**Published:** 2023-06-27

**Authors:** Valentina Venturi, Francesco Presini, Claudio Trapella, Olga Bortolini, Pier Paolo Giovannini, Lindomar Alberto Lerin

**Affiliations:** 1 Department of Environment and Prevention Sciences, University of Ferrara – UNIFE, Via Luigi Borsari, 46, Ferrara, 44121 Italy; 2 Department of Chemical, Pharmaceutical and Agricultural Sciences, University of Ferrara – UNIFE, Via Luigi Borsari, 46, Ferrara, 44121 Italy

**Keywords:** Geranyl acetoacetate, Geranyl (*R*)-3-hydroxybutyrate, Geranyl butyrate, Geranyl octanoate, Geranyl hexanoate, Lipozyme 435

## Abstract

Various geraniol esters act as insect pheromones and display pharmacological activities, especially as neuroprotective agents. Therefore, the search for synthetic strategies alternative to traditional chemical synthesis could help designing ecofriendly routes for the preparation of such bioactive compounds. Hence, this work aims at the microwave-assisted enzymatic synthesis of geranyl esters in solvent-free systems. The process variables were optimized for the synthesis of geranyl acetoacetate, achieving 85% conversion after 60 min using a 1:5 substrates molar ratio (ester to geraniol), 80 °C and 8.4% of Lipozyme 435 lipase without removal of the co-produced methanol. On the other hand, a 95% conversion was reached after 30 min using 1:6 substrates molar ratio, 70 °C and 7% lipase in the presence of 5Å molecular sieves for the methanol capture. In addition, the lipase showed good reusability, maintaining the same activity for five reaction cycles. Finally, under the above optimized conditions, other geraniol esters were successfully synthetized such as the geranyl butyrate (98%), geranyl hexanoate (99%), geranyl octanoate (98%), and geranyl (*R*)-3-hydroxybutyrate (56%). These results demonstrate the microwave-assisted lipase-catalyzed transesterification in a solvent-free system as an excellent and sustainable catalytic methodology to produce geraniol esters.

**Graphical Abstract:**

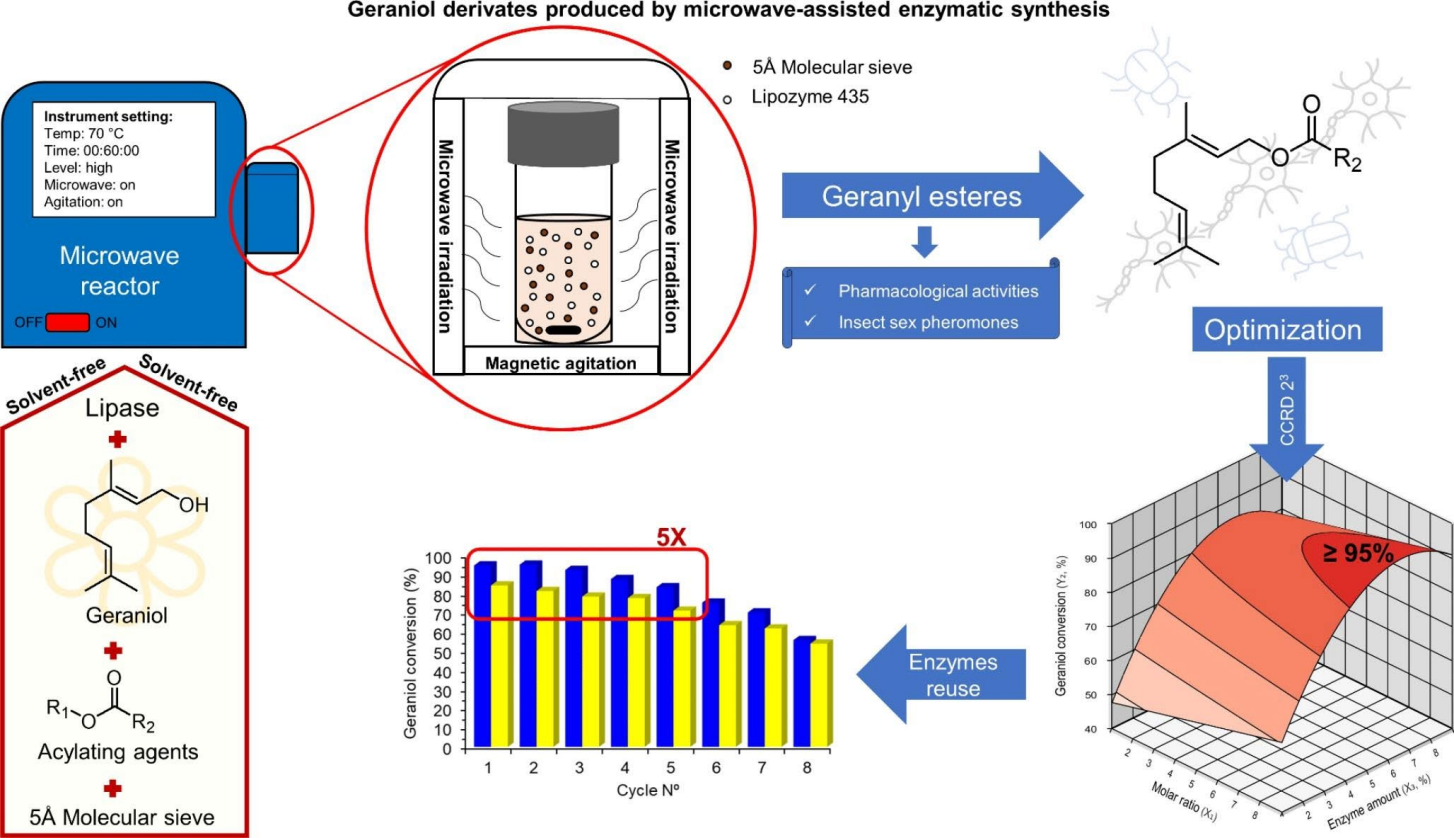

**Supplementary Information:**

The online version contains supplementary material available at 10.1007/s11030-023-10682-y.

## Introduction

Primary and secondary plant metabolites, such as essential oils, are important building blocks for synthesizing new bio-based compounds with various applications. Essential oils are complex mixture of secondary plant metabolites represented by aromatic, aliphatic, and volatile liquid compounds which have been extensively examined for their pharmacological activities [1]. Among these compounds, geraniol (*trans*-3,7-dimethyl-2,6-octadien-1-ol) is a primary, acyclic, doubly unsaturated monoterpene alcohol, abundant in various essential oils from plants such as rose, lemongrass, lavender, palmarosa, geranium, and others, that in virtue of its floral odor, like rose, is widely used as a flavoring agent in cosmetics and perfumes [2, 3]. The pharmacological properties described for geraniol are antioxidant, anti-inflammatory, immunostimulant, antimicrobial, and recent studies have demonstrated neuroprotective properties as well [1–5]. These last include the promotion of survival of dopaminergic neurons and positive effects in the treatment of Parkinson’s disease [6–9].

Various geraniol esters have been extracted from plant matrices [10–12]. However, its synthetic production seems more convenient for industrial processes as it allows the control of process conditions and presents high yields. It also allows to use heterogeneous catalysts that are easily separable from the reaction medium, non-corrosive, reusable and easy to handle and store [5, 13–15]. Recent studies highlighted the neuroprotective properties of geranyl ursodeoxycholate and geranyl acetate [9, 16]. Instead, several aliphatic and aromatic esters of geraniol, such as the octanoate, butanoate, isovalerate, hexanoate, propionate, acetate, nonanoate, valerate, acetoacetate, and benzoate, showed activity as insect pheromones [17–22].

Other natural compounds that have drawn attention in recent years for their wide range of biological activities are the ketone bodies, represented by (*R*)-3-hydroxybutyrate (3HB), acetoacetate (AcAc), and acetone. The human body produces these compounds as a survival mechanism to provide energy substrates for the brain and skeletal muscles during exercise in a fasted state [23, 24]. Recent research indicates that exogenous ketone therapy can preserve or improve motor and cognitive performance [23, 25–28]. In addition, several studies suggest that diets supplemented with exogenous ketone bodies may have therapeutic effects for the prevention and/or treatment of neurological pathologies such as epilepsy, Parkinson’s and Alzheimer’s disease, and psychiatric disorders [24, 29–32]. The current trend is to administer the ketone bodies 3HB and AcAc under an esterified form rather than as free acids or the corresponding salts in order to reduce blood acidosis or cations accumulation, respectively [33]. Esters such as (*R*/*S*)-3-hydroxybutyl acetoacetate [25, 34, 35], (*R*)-3-hydroxybutyl (*R*)-3-hydroxybutyrate [33, 36, 37], hexyl (*R*)-3-*O*-hexanoyl-3-hydroxybutyrate [38], glyceryl acetoacetate and glyceryl-3-hydroxybutyrate [39] have been used to induce ketosis.

Esters are traditionally synthesized using acidic or basic catalysts in the presence or not of ester coupling reagents. Esters of geraniol with ursodeoxycholic and acetic acid, as well as esters of acetoacetic acid with glycerol or other alcohols (menthyl, 3-phenylpropyl and benzyl), have been synthesized through traditional chemical routes [9, 13, 40–42]. However, eco-sustainable catalytic methodologies, such as biocatalysis, are being used to replace chemical catalysis. Biocatalysis is considered one of the main drivers of innovation in organic synthesis [43–45], either in the academy or industry. The most used enzymes in bioprocesses are the lipases (triacylglycerol acyl hydrolases, EC 3.1.1.3), which catalyze reactions such as hydrolysis, esterification, transesterification, and interesterification, in addition to the so-called promiscuous reactions and are stable in a wide range of media [45–52]. Lipases have many advantages, such as normally showing high catalytic efficiency and excellent chemo-, regio- and stereoselectivity. These features allow high yield with reduced by-product formation, resulting in the simplest purification and downstream phases. In addition, the mild reaction conditions usually employed and the possibility of reusing immobilized enzymes contribute to the economic and environmental sustainability of biocatalyzed productive processes [53]. Lipases are generally stable in a wide range of media and, as immobilized form, have been used in various reactor models such as stirred tank reactors (STR), packed bed reactors (PBR), and fluidized bed reactors (FBR) with different configurations and operation methods, which may be associated with technologies suited to intensify the enzymatic reactions such as microwave, ultrasound, and supercritical fluids [46, 54–56].

Microwave technology has established itself as an efficient heating source that can increase conversion and yield in shorter reaction times, thus reducing energy consumption compared to other heating techniques. Therefore, microwaves have been applied to various synthetic reactions, including enzyme-catalyzed ones [56, 57]. In addition, microwave-assisted syntheses allow better performance for reactions carried in the absence of solvent, which often afford high-purity products with easier downstream, reducing the process’s operating costs [54]. Due to these characteristics, microwaves irradiation is considered a suited technology for process intensification [58–60]. Also, it can improve enzyme stability by delaying denaturation in comparison with conventional heating systems, as demonstrated for the lipase B from *Candida antarctica* [56, 60]. The literature is abundant in describing microwave-assisted enzymatic synthesis of esters [54, 56, 60, 61], among which are geraniol esters such as geranyl butyrate [62] and geranyl cinnamate [57]; and acetoacetic acid esters such as propyl acetoacetate, butyl acetoacetate, pentyl acetoacetate, hexyl acetoacetate, octyl acetoacetate, and decyl acetoacetate [63].

**Fig. 1:**
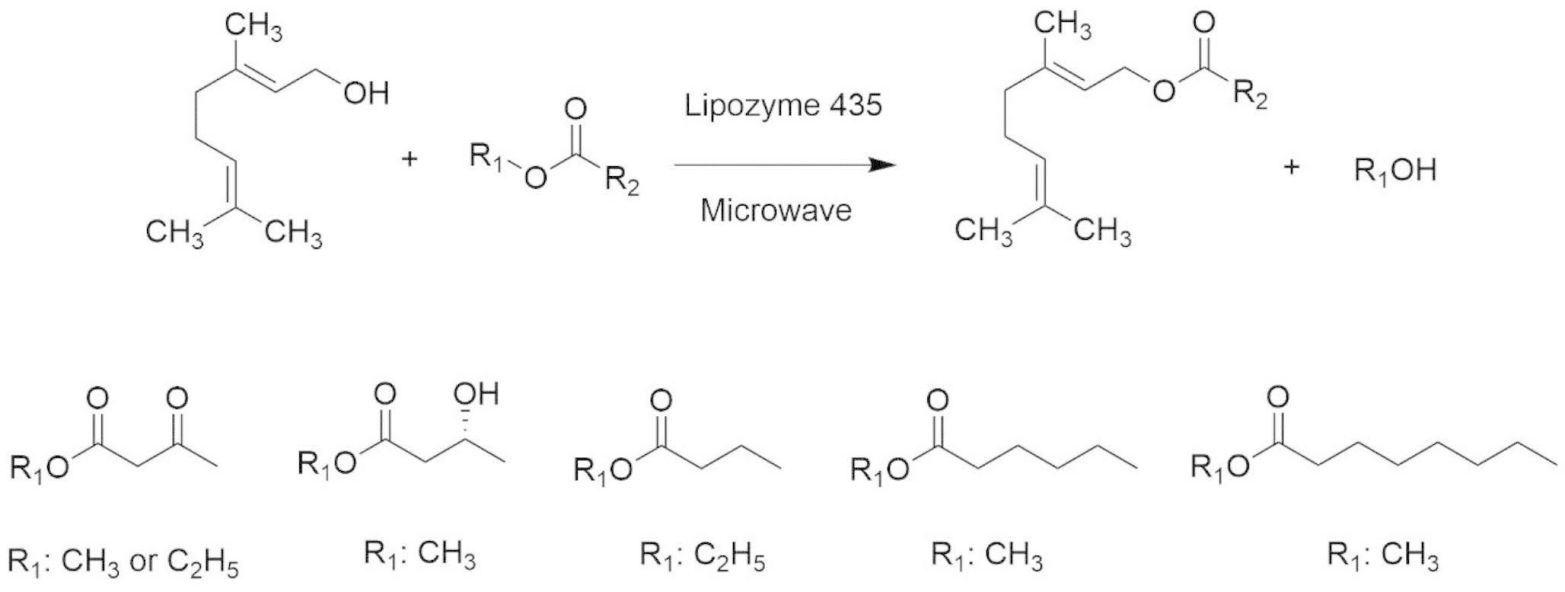
Scheme of the Lipozyme 435 catalyzed synthesis of geranyl esters and structures of the acylating agents employed

In this context, we reported the transesterification of methyl acetoacetate with geraniol promoted by the lipase B from *Candida antarctica* (Lipozyme 435). In addition, a comparative study of this enzymatic reaction performed under conventional heating or microwave irradiation in an STR has been conducted. Furthermore, the microwave-assisted reaction has been optimized through a Central Composite Rotatable Design (CCRD) by considering the substrates molar ratio, the temperature, and the enzyme amount as the independent variables. Finally, the synthesis of geranyl butyrate, geranyl octanoate, geranyl hexanoate, and geranyl (*R*)-3-hydroxybutyrate have been explored under the conditions optimized for the synthesis of the geranyl acetoacetate (Fig. 1). From the literature review, the synthesis of geranyl acetoacetate, geranyl hexanoate, and geranyl (*R*)-3-hydroxybutyrate by enzymatic route was not found.

## Experimental methodology

### Chemicals and enzymes

Geraniol (97% purity), methyl acetoacetate (97% purity), ethyl acetoacetate (99% purity), methyl hexanoate (99% purity), methyl octanoate (99% purity), ethyl butyrate (99% purity), and methyl (*R*)-3-hydroxybutyrate (99% purity) were purchased from Sigma-Aldrich. The lipase B from *Candida antarctica* immobilized on acrylic resin was purchased from Novozymes SA under the commercial name of Lipozyme 435 (declared activity 9000 PLU g^− 1^). Molecular sieve beads 5 Å (8–12 mesh) purchased from Sigma-Aldrich. Ethyl acetate (Carlo Erba), cyclohexane (Carlo Erba), acetic acid (Sigma-Aldrich), acetone (Carlo Erba), and deuterated chloroform (Sigma-Aldrich) were of analytical grade and used as such without further purification.

### Gas-chromatographic analysis

The reaction sample was filtered (filter paper), centrifuged (14,000 rpm for 2 min), and diluted with ethyl acetate (1:100) before the gas chromatographic analysis. The analysis was performed on a Thermo Focus GC with a flame ionization detector (FID) and a MEGA-S52 capillary column from MEGA S.r.l. (30 m length x 0.32 mm internal diameter x 0.1–0.15 μm film thickness). The oven temperature program was: 100 °C for 2 min, then raised to 230 °C with a rate of 10 °C min^− 1^. Injector and detector temperatures were maintained at 175 and 250 °C, respectively. The injection mode selected was the split ratio (1:50), and a volume of 1 µL was injected, using He as the carrier gas. The conversions were calculated based on the ratio between the peak area of the product and that of the limiting reagent (geraniol) [14, 15]. Retention times: geranyl acetoacetate 11.6 min; geranyl-3-hydroxybutyrate 11.5 min; geranyl octanoate 14.1 min; geranyl hexanoate 12.1 min; and geranyl butyrate 10.0 min.

### Microwave reactor

Microwave-assisted enzymatic reactions were carried out using a Biotage Initiator™ with a single-mode cavity dedicated reactor equipped with an external infrared (IR) sensor for temperature control, magnetic stirring, absorption level (normal, high, and very high) control, and a non-invasive pressure sensor integrated into the cavity reactor lid. Glass reactors with 2 to 5 mL volume sealed with Teflon septum and aluminum crimp (using an appropriate crimping tool) were used.

### Optimization of the microwave-assisted geranyl acetoacetate (GAcAc) synthesis with and without 5Å molecular sieves

Microwave-assisted GAcAc synthesis was maximized through the experimental design (DOE) methodology [64]. A 2^3^ CCRD design was used, considering two levels (-1 and + 1) and triplicate of the central point (0). The independent variables evaluated were the molar ratio of geraniol to methyl acetoacetate (mmol/mmol), the temperature (°C), and the enzyme amount (percentage of the biocatalyst to the geraniol mass). In contrast, the geraniol conversion to GAcAC (%) was considered the dependent variable or response. All the reactions were conducted in batch mode using Lipozyme 435 (Lipo 435) as the biocatalyst. A magnetic pre-agitation of 30 s was adopted to homogenize the substrates, and, during the reactions, the stirring, and the absorption level (high) were kept constant. The coded and real values of the independent variables are reported in Table 1.

The reactions were performed, with or without 5Å molecular sieve (5Å MS). The 5Å MS amounts added were calculated based on methanol-absorbent capacity (200 mg.g^− 1^), considering the theoretical maximum amount of methanol produced in each reaction.

**Table 1:** Matrix of 2^3^ CCRD (coded and real values) and responses in terms of geraniol conversion in percentage using the Lipozyme 435 without and with 5Å MS

Trial	RM^1^	T (°C)	E (wt%)^2^	Lipozyme 435			Lipozyme 435–5Å MS			
				Experimental (%)	Predicted^3^ (%)	RE^4^ (%)		Experimental (%)	Predicted^3^ (%)	RE^4^ (%)
1	-1 (1:3)	-1 (60)	-1 (3)	53	49.3	6.8		74	72.0	3.9
2	+ 1 (1:7)	-1 (60)	-1 (3)	51	49.3	3.2		79	78.4	0.7
3	-1 (1:3)	+ 1 (80)	-1 (3)	64	57.5	10.0		77	72.0	6.4
4	+ 1 (1:7)	+ 1 (80)	-1 (3)	61	57.5	5.6		80	78.4	1.9
5	-1 (1:3)	-1 (60)	+ 1 (7)	66	68.2	-3.3		86	87.3	-1.5
6	+ 1 (1:7)	-1 (60)	+ 1 (7)	71	68.2	3.9		90	93.7	-4.1
7	-1 (1:3)	+ 1 (80)	+ 1 (7)	73	76.4	-4.7		90	87.3	2.9
8	+ 1 (1:7)	+ 1 (80)	+ 1 (7)	79	76.4	3.2		93	93.7	-0.7
9	-1.68 (1:1.6)	0 (70)	0 (5)	56	65.5	-16.9		75	82.0	-9.4
10	+ 1.68 (1:8.4)	0 (70)	0 (5)	67	65.5	2.2		92	92.7	-0.8
11	0 (1:5)	-1.68 (56)	0 (5)	58	58.6	-1.0		86	87.4	-1.6
12	0 (1:5)	+ 1.68 (87)	0 (5)	70	72.4	-3.4		91	87.4	3.9
13	0 (1:5)	0 (70)	-1.68 (1.6)	35	42.2	-20.8		57	61.7	-8.3
14	0 (1:5)	0 (70)	+ 1.68 (8.4)	76	74.0	2.6		90	87.4	2.8
15	0 (1:5)	0 (70)	0 (5)	67	65.5	2.2		87	87.4	-0.4
16	0 (1:5)	0 (70)	0 (5)	65	65.5	0.7		88	87.4	0.6
17	0 (1:5)	0 (70)	0 (5)	66	65.5	-0.7		89	87.4	1.7

^1^ Molar ratio geraniol to methyl acetoacetate

^2 ^% biocatalyst to the geraniol mass

^3^ Calculated according to Eq. 1 e 2

^4^ $$Relative\, Error \left(RE, \%\right)=\left(\frac{Experimental conversion-Predicted\, conversion}{Experimental\, conversion}\right)*100$$

**Fig. 2:**
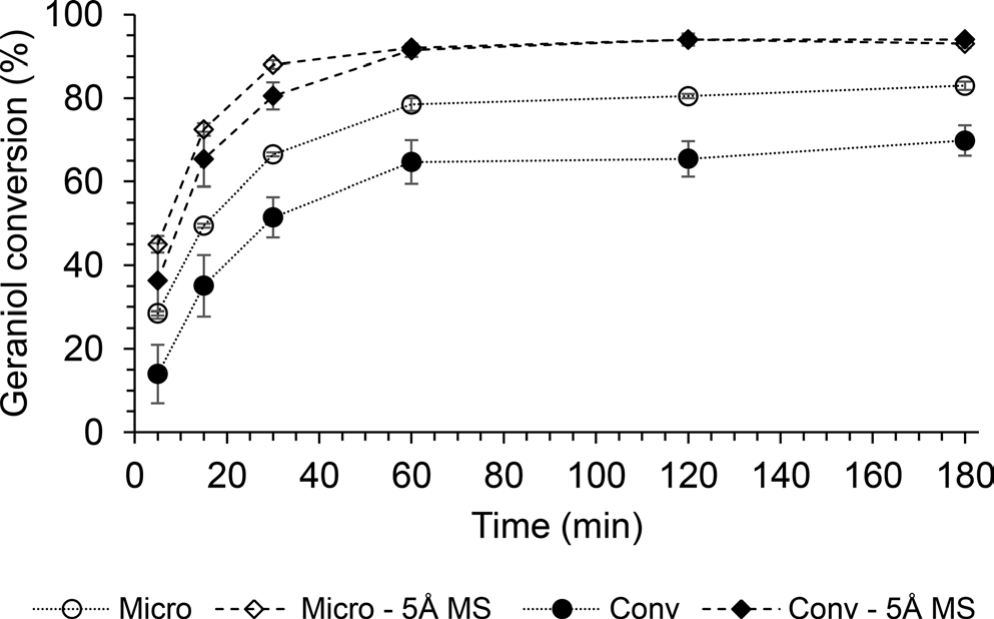
Kinetics of the microwave-assisted (Micro) and conventional (Conv) heated GAcAc enzymatic synthesis with and without methanol removal (5Å MS). Reactional conditions as for the central point of the DOE (Table 1, trials 15–17)

The reactions performed without 5Å MS (1.5 mL total volume) were conducted in a 5 mL conical-bottom reactor, whereas the reactions with 5Å MS (total volume of 2 mL) were performed in a 5 mL concave-bottom reactor. The reaction starting time was considered one minute after the beginning of microwave irradiation (time required to reach the programmed temperature). A 30 min reaction time was chosen for the CCRD trials based on the kinetic study previously performed with and without 5Å MS (Fig. 2) in the central point conditions (Table 1, trials 15–17). Blanc experiments in the absence of Lipo 435 were carried out for all the conditions studied with a 180 min reaction time. In any case, the production of geranyl acetoacetate was not observed (data not shown). At the end of each reaction, the mixture was treated as reported above for the GC analysis.

The online software Protimiza Experimental Design (http://experimentaldesign.protimiza.com.br/) was used to assist the design and statistical analysis of the DOE, adopting a significance level of 90% (*p* ≤ 0.1) in both reaction systems.

### CCRD validation for the microwave-assisted synthesis of GAcAc without and with 5Å MS

To validate the CCRD the effect of the temperature was investigated in the absence of 5Å MS by performing the reaction at four different temperatures using the same molar ratio and enzyme amount (Table 2). On the other hand, the effects of the substrate’s molar ratio and the enzyme amount were evaluated in the presence of 5Å MS according to the conditions described in Table 2. Five reactions were performed for each condition, either in the presence or absence of 5Å MS, by stopping the microwave irradiation after 5, 15, 30, 60, and 120 min. The reaction mixtures were treated as above reported and analyzed by GC.

**Table 2:** Experimental conditions used to validate the CCRD without and with 5Å MS

MR^1^	T (°C)	E (wt%)^2^
Without 5Å MS		
1:5	70	8.4
	75	
	80	
	85	
With 5Å MS		
1:1	70	7
1:3		
1:4		
1:6		
1:3	70	1
		3
		5
		7

^1^ Molar ratio geraniol to methyl acetoacetate

^2 ^% biocatalyst to the geraniol mass

### Enzyme reusability in the microwave-assisted GAcAc synthesis without and with 5Å MS

The reusability of the immobilized enzyme was investigated in the presence or not of 5Å MS under the optimized conditions for both systems, namely a substrate molar ratio of 1:3, a temperature of 70 °C, and 7% of the enzyme in the presence of 5Å MS, and a molar ratio of 1:5, a temperature of 80 °C, and 8.4% of the enzyme in the absence of 5Å MS. The reactions were irradiated for 1 h and then submitted to a double filtration, the first using a 7-mesh filter to remove the 5Å MS and the second using filter paper to recover the enzyme. The recovered enzyme was washed with acetone and dried under vacuum (30 mmHg) in a rotary evaporator at 40 °C for 20 min. The dried enzyme was used for the following reaction cycle. Enzyme performance was evaluated regarding geraniol conversion to GAcAc obtained in each cycle. All reactions were performed in duplicate.

### Microwave-assisted geranyl esters synthesis

The microwave-assisted transesterification of the alkyl esters of different acids (ethyl acetoacetate, ethyl butyrate, methyl hexanoate, methyl octanoate, methyl (*R*)-3-hydroxybutyrate) with geraniol was evaluated under the condition optimized for the GAcAc production. All the reactions were performed in the presence of 5Å MS, using a 1:3 molar ratio (geraniol to ester), 70 °C, and 3% of the enzyme by stopping the microwave irradiation after 5, 15, 30, 60, and 120 min. The reaction mixtures were treated as above reported and analyzed by GC.

### Conventionally heated GAcAc synthesis

Reaction kinetics with conventional heating mode using a thermostatic bath was performed under the condition of the central point of the CCRD (Table 1, trials 15–17) with or without 5Å MS using the identical reactors and reaction mixture volumes reported in item 2.4. The reaction mixtures were treated and analyzed as above.

### Geranyl esters purification and spectroscopic characterization

After removal of the enzyme and 5Å MS, the reaction mixture was evaporated under reduced pressure. The residue was chromatographed on silica gel (60 Å, 70–230 mesh, particle size 63–200 μm; Sigma-Aldrich) using cyclohexane/ethyl acetate (15:1) added with acid acetic (0.2%) as the eluent. The fractions containing the product were identified by TLC analyses performed on silica gel 60 F254 with detection by charring with phosphomolybdic acid. The purified products ^1^ H and ^13^ C NMR spectra were acquired at room temperature on a spectrometer operating at 400 MHz, using CDCl_3_ as the solvent.

*Geranyl acetoacetate* (GAcAc) - ^1^ H NMR (400 MHz, CDCl_3_) δ 5.37–5.31 (m, 1 H), 5.10–5.04 (m, 1 H), 4.66 (d, *J* = 7.2 Hz, 2 H), 3.44 (s, 2 H), 2.26 (s, 3 H), 2.15-2.00 (m, 4 H), 1.70 (s, 3 H), 1.67 (s, 3 H), 1.59 (s, 3 H). ^13^ C NMR (101 MHz, CDCl_3_) δ 200.6, 167.2, 143.1, 131.9, 123.6, 117.6, 62.2, 50.1, 39.5, 30.1, 26.2, 25.7, 17.7, 16.5.

*Geranyl (R)-3-hydroxybutyrate* (G-3HB) - ^1^ H NMR (400 MHz, CDCl_3_) δ 5.36–5.30 (m, 1 H), 5.10–5.04 (m, 1 H), 4.63 (d, *J* = 7.2 Hz, 2 H), 4.25–4.10 (m, 1 H) 3.03 (br s, 1 H), 2.49 (dd, *J* = 16.5, 3.5 Hz, 1 H), 2.41 (dd, *J* = 16.5, 8.6 Hz, 1 H), 2.14–2.00 (m, 4 H), 1.70 (s, 3 H), 1.67 (s, 3 H), 1.59 (s, 3 H), 1.22 (d, *J* = 6.3 Hz, 2 H). ^13^ C NMR (101 MHz, CDCl_3_) δ 172.9, 142.8, 131.9, 123.6, 117.9, 64.2, 61.5, 42.7, 39.5, 26.2, 25.6, 22.3, 17.7, 16.4.

*Geranyl butyrate* (GB) - ^1^ H NMR (400 MHz, CDCl_3_) δ 5.37–5.30 (m, 1 H), 5.10–5.04 (m, 1 H), 4.59 (d, *J* = 7.1 Hz, 2 H), 2.28 (t, *J* = 7.4 Hz, 2 H), 2.15-2.00 (m, 4 H), 1.70 (s, 3 H), 1.68–1.61 (m, 2 H), 1.67 (s, 3 H), 1.59 (s, 3 H), 0.94 (t, *J* = 7.4 Hz, 3 H). ^13^ C NMR (101 MHz, CDCl_3_) δ 173.7, 142.1, 131.8, 123.7, 118.4, 61.1, 39.5, 36.25, 26.3, 25.6, 18.5, 17.6, 16.4, 13.6.

*Geranyl hexanoate* (GH) - ^1^ H NMR (400 MHz, CDCl_3_) δ 5.36–5.29 (m, 1 H), 5.11–5.04 (m, 1 H), 4.58 (d, *J* = 7.1 Hz, 2 H), 2.29 (t, *J* = 7.6 Hz, 2 H), 2.14–2.00 (m, 4 H), 1.70 (s, 3 H), 1.68–1.58 (m, 2 H), 1.68 (s, 3 H), 1.59 (s, 3 H), 1.36–1.23 (m, 4 H), 0.89 (t, *J* = 7.0 Hz, 3 H). ^13^ C NMR (101 MHz, CDCl_3_) δ 173.9, 142.1, 131.8, 123.7, 118.4, 61.1, 39.5, 34.3, 31.3, 26.3, 25.6, 24.7, 22.3, 17.6, 16.4, 13.9.

*Geranyl octanoate* (GO) - ^1^ H NMR (400 MHz, CDCl_3_) δ 5.37–5.30 (m, 1 H), 5.11–5.05 (m, 1 H), 4.58 (d, *J* = 7.1 Hz, 2 H), 2.29 (t, *J* = 7.7 Hz, 2 H), 2.15–2.00 (m, 4 H), 1.70 (s, 3 H), 1.68–1.57 (m, 2 H), 1.68 (s, 3 H), 1.60 (s, 3 H), 1.35–1.20 (m, 8 H), 0.87 (t, *J* = 6.9 Hz, 3 H). ^13^ C NMR (101 MHz, CDCl_3_) δ 173.9, 142.1, 131.8, 123.7, 118.4, 61.1, 39.5, 34.4, 31.6, 29.1, 28.9, 26.3, 25.7, 25.0, 22.6, 17.7, 16.4, 14.0.

## Results and discussion

### Microwave versus conventional GAcAc enzymatic synthesis

The increase in temperature leads to higher energy collisions with the resulting increase in reaction rates. This result can be achieved much faster with microwave irradiation due to instantaneous heating. The literature states that many enzymatic reactions afford higher yields in shorter times when conducted under microwave irradiation [56, 57, 62]. Therefore, the enzymatic transesterification of methyl AcAc with geraniol was studied either under microwave irradiation or conventional heating. The reactions were catalyzed by the Lipo 435, and the effect of the addition of 5Å MS for the methanol removal was evaluated as well. As shown in Fig. 2, conversion values near to the maximum were reached within 60 min followed by little increments in the next two ours, regardless of the type of heating and the presence or not of 5Å MS. The conventionally heated reactions reached the maximum conversion values of 94 and 70% after 180 min in the presence or not of 5Å MS, respectively. Thus, removing methanol provided a 26% increase in conversion after 60 min, which became 24% after 180 min. However, both reactions showed a slow increase in the conversion from 60 to 180 min (about 5%).

A similar increase in the maximum conversion was observed also for the microwave-assisted reactions, when 5Å MS were used for the removal of released methanol (from 83 to 94% after 180 min).

The positive effect of microwave irradiation on the reaction rate is evident from comparing the conversion values measured after the first five minutes. These results showed that this effect is even more apparent in the absence of 5Å MS, with a 62% increase in conversion between microwave-assisted and conventionally heated reactions. As regards the reactions carried out in the presence of 5Å MS, the microwave-assisted ones showed a conversion increase of 39% after 5 min with respect to the conventionally heated. This behavior accords to what observed by several authors who reported higher initial reaction rates for microwave-assisted enzymatic reactions compared to the corresponding conventionally heated [57, 63].

For reaction times longer than 60 min, the two heating systems afforded similar results in the presence of 5Å MS. On the contrary, in the absence of methanol removal systems, the microwave-assisted reaction showed better performances with an average gain in the conversion of approximately 13% (Fig. 2).

Based on these results, the optimization study was continued using the Lipo 435 catalyzed transesterification of methyl acetoacetate with geraniol performed under microwave heating either in the presence or not of 5Å MS for the removing of the co-produced methanol.

### Optimization of the microwave-assisted GAcAc synthesis by CCRD

The microwave-assisted enzymatic transesterification of methyl AcAc with geraniol in a solvent-free system was maximized through CCRD by considering the substrates molar ratio (geraniol to methyl acetoacetate), the temperature, and the enzyme amount as the independent variables. Based on the kinetics reported in Fig. 2, a reaction time of 30 min was chosen for the 2^3^ CCRD. Table 1 shows the 2^3^ CCRD matrix with the coded and real values for the independent variables (columns 2–4), the experimentally determined and predicted response in terms of geraniol conversion to GAcAc with the corresponding relative error, either for the reactions performed with or without 5Å MS (columns 5–7 and 8–10, respectively). As expected, higher conversions, with values greater than 90%, were obtained when the methanol formed was sequestered from the reaction mixture by the 5Å MS. The maximum conversion value of 93% was obtained with the methanol removal system against 79%, showed by the analogue reaction performed in the absence of 5Å MS (Table 1, trial 8).

The results obtained in the CCRD were statistically treated by analysis of variance (ANOVA) to verify the goodness of the empirical model in reproducing the experimental data for the GAcAc production. The pure error found in evaluating the CCRD results is low. It tends toward zero [(2/1537.7)x100 = 0.13% without 5Å MS and (2/1207.6)x100 = 0.16% with 5Å MS, respectively] confirming the good experimental reproducibility of the tests, which is a consequence of the excellent reproducibility of the tests carried out at the central point (Table 1, trials 15 to 17). The analysis of the *F* test (*p* ≤ 0.1) shows that the *F*_calc_ is 10.07 and 14.45 times greater than the *F*_tab_ for the reactions performed with and without 5Å MS, respectively, thus indicating that there are significant differences between the means. The percentage of the total variance model was also evaluated through R^2^, presenting values of 86 and 89% for the systems with and without 5Å MS, respectively (Table 3). Confirmation of this conclusion can be seen in Table 1, which shows as the conversion values predicted by Eqs. 1 and 2 excellently fit with the experimental data with very low relative errors found.

**Fig. 3:**
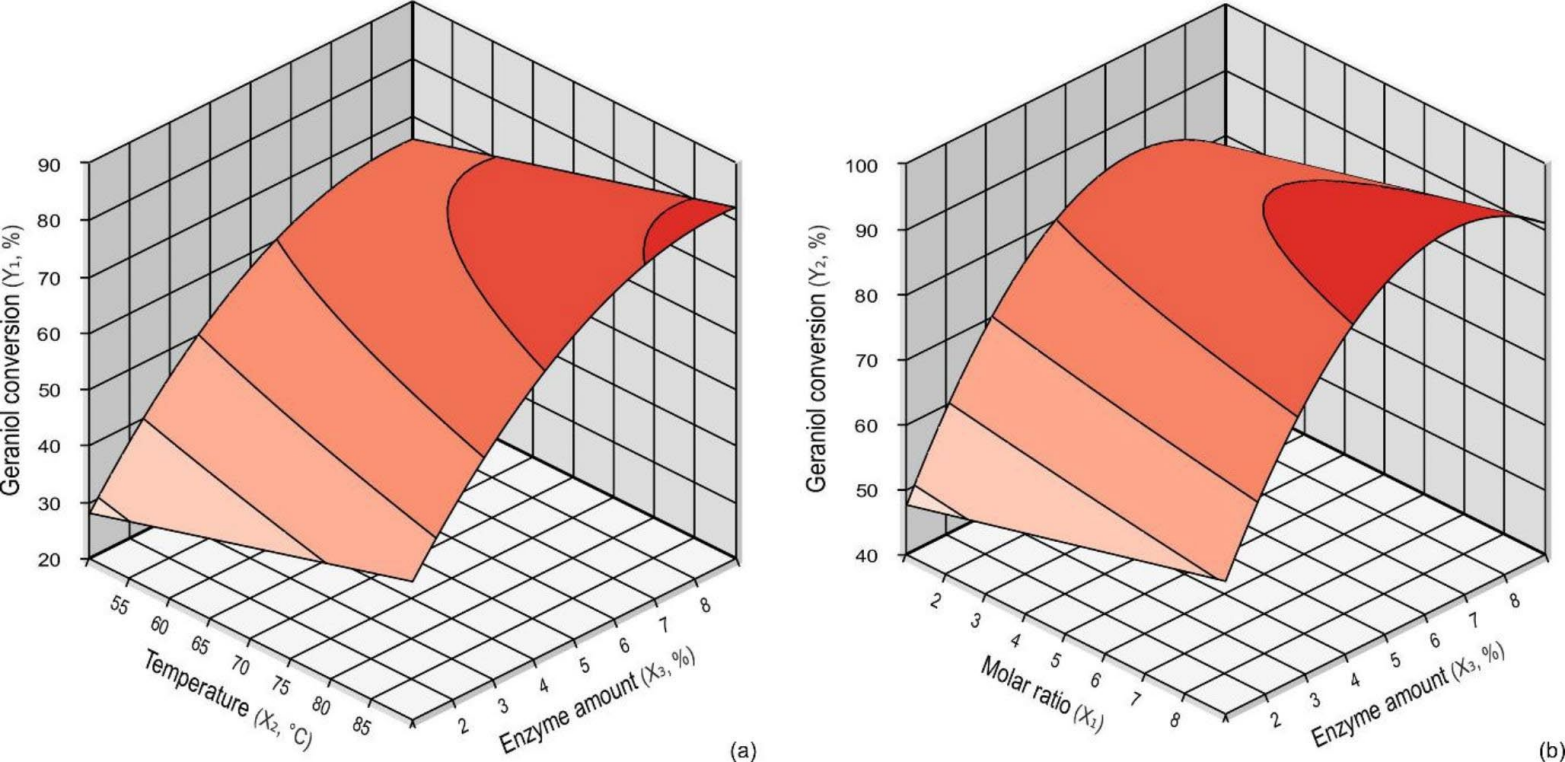
Response surface for microwave-assisted geraniol conversion without (**a**) and with (**b**) 5Å MS as a function of significant interaction of independent variables

Equations 1 and 2 also allowed the construction of response surfaces (Fig. 3) from which the effect of independent variables on the conversion of geraniol to GAcAc can be deduced. These studies showed that the temperature and the enzyme amount were the variables that mainly affected the conversion when the methanol was not removed (Table 3, Eq. 1), while, in the presence of 5Å MS, the substrates molar ratio and the enzyme amount were the variables with the more significant effect on the conversion (Table 3, Eq. 2). The enzyme amount was the variable with the greatest positive significant effect in both the systems in terms of its linear function, as can be seen from trials 6 to 8 (Table 1), which showed conversions higher than 70% and 90% without and with methanol removal, respectively. However, the quadratic function (Eqs. 1 and 2) showed a significant negative effect, as can be seen in the star points, where the conversion achieved with the highest enzyme amount of 8.4% (trial 14, Table 1) showed slightly lower conversions (76 and 90%) concerning the maximum values (79 and 93%) obtained with 7% of enzyme amount (Table 1, trial 8). Shinde and Yadav have reported a similar behavior in synthesizing geranyl cinnamate. They attributed the low significant gains in conversion observed with the higher amounts of the enzyme to diffusion resistance phenomena due to the excess of available active sites [57]. The effect of the enzyme amount on the conversion is deductible from Fig. 3a and b, where the regions of maximum conversion coincide with the enzyme amount comprising 7 and 8% for both reaction systems.

The temperature had a significant positive effect only for reactions performed without 5Å MS. In these reactions, temperature increases determined corresponding increases in the conversion, as can be observed in Fig. 3a, where the region of the maximum conversions is in the highest temperature range (above 80 °C). Shinde and Yadav have also described similar behavior in the above-cited study [57]. These results can be explained by assuming that higher temperatures favor the methanol evaporation with the consequent lowering of its concentration in the reaction mixture. This hypothesis is corroborated by the absence of a similar positive effect of the temperature in the reactions carried out with 5Å MS.

For reactions performed with 5Å MS, the reagent’s molar ratio and the enzyme amount were the variable that significantly positively affected the conversion (Table 3, Eq. 2). The highest conversions obtained in this study (93 and 92%) were obtained with the highest molar ratios employed (Table 1, trials 8 and 10). Figure 3b confirms this, showing a region of maximum conversion comprised in the range of the highest molar ratios. A similar effect was reported for the enzymatic transesterification of methyl acetoacetate with *n*-butanol in a solvent-free system [63]. It can be explained by considering that under this condition, the excess of a substrate (ester) plays an essential role in reaction displacement, mass transfer, and solubilization phenomena because of its additional role as a solvent [54, 65].

**Table 3:** Percentage of explained variance (R^2^), calculated *F* (*F*_calc_.) value, tabulated *F* (*F*_tab_.) value, and *F*_calc_./*F*_tab_. for the responses geraniol conversion, by analysis of variance (ANOVA). Statistically significant models have F_calc_./F_tab_. values greater than 1

Response model	Equation	R^2^ (%)	*F*_calc_.	*F*_tab_.	$\frac{{F}_{calc.}}{{F}_{tab.}}$
${y}_{1}=65.51+4.11T+9.44E-2.61{E}^{2}$	1	86	25.79	2.56	10.07
${y}_{2}=87.41+3.19MR+7.65E-4.54{E}^{2}$	2	89	34.54	2.56	14.49

*y*_*1*_ is the % conversion without 5Å MS (%), *y*_*2*_ is the % conversion with 5Å MS, *T* is temperature (°C), *E* is enzyme amount (%), and *MR* is substrate molar ratio

From the analysis of the CCRD results, it can be stated that the order of the effects of the independent variables on the microwave-assisted conversion of geraniol to GAcAc can be classified as enzyme amount > temperature > substrates molar ratio for the system without 5Å MS, which is in agreement with that reported for the microwave-assisted enzymatic synthesis of geranyl cinnamate without the removing of the co-produced ethanol [57]. In contrast, the order for the system with 5Å MS was enzyme amount > molar ratio > temperature. The enzyme amount had a great effect under both the reaction systems because, in solvent-free reactions, the high concentration of the reagents magnifies their potential inhibitory effects, which are even more evident when smaller amounts of the enzyme are used [65].

In summary, the optimal conditions for the microwave-assisted lipase-catalyzed conversion of geraniol to GAcAc, either without or with 5Å MS, were those reported for trial 8 in Table 1, namely, substrates molar ratio of 1:7, the temperature of 80 °C, and enzyme amount of 7%. Under these conditions, conversions of 79 and 93% were achieved after 30 min in the absence or in the presence of 5Å MS for the methanol removal, respectively.

### CCRD validation for enzymatic microwave-assisted GAcAc synthesis without and with 5Å MS

**Fig. 4:**
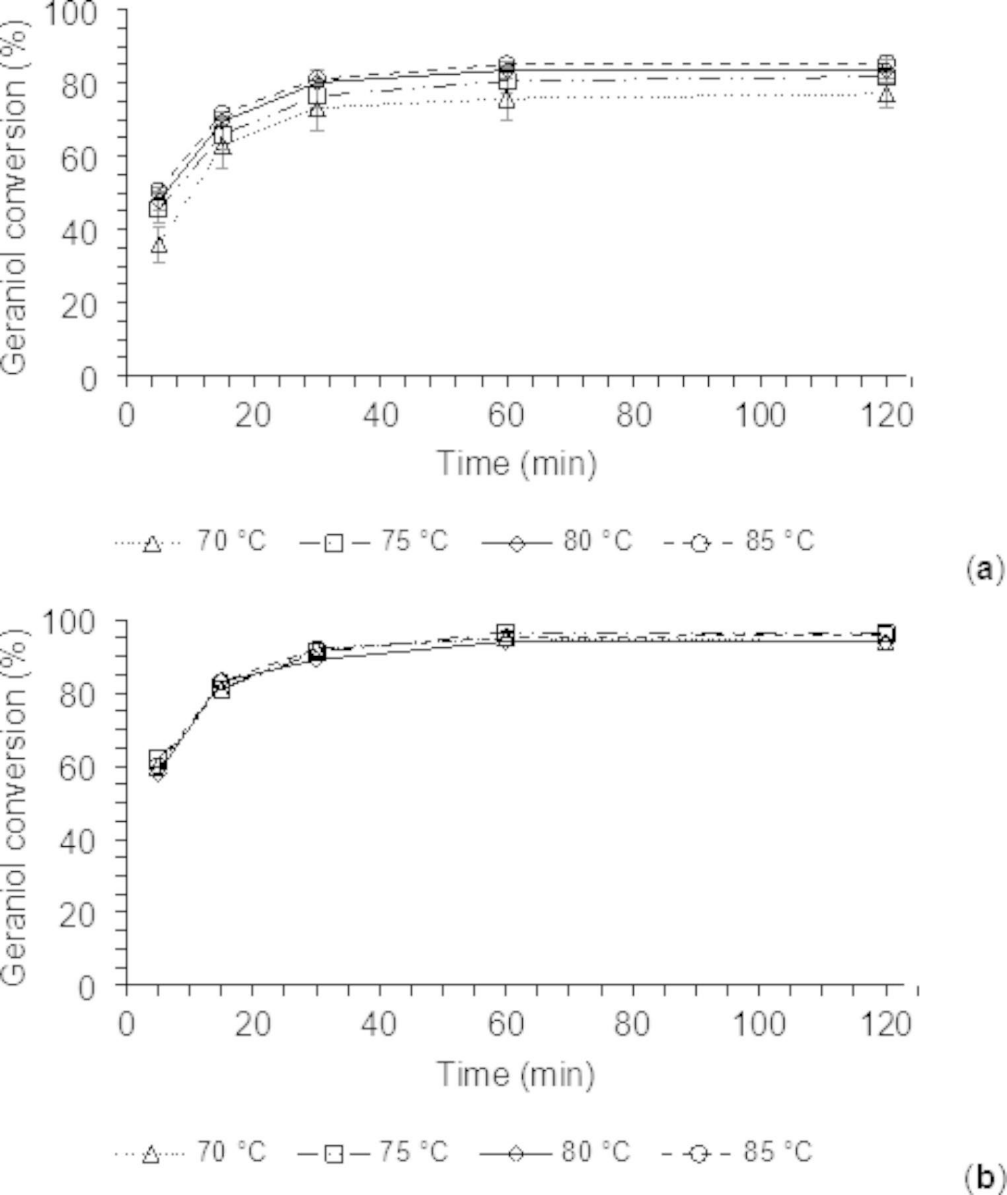
Kinetics of experimental validation of the temperature condition for the microwave-assisted enzymatic synthesis of GAcAc without (**a**) and with (**b**) 5Å MS

A kinetic study for the microwave-assisted enzymatic synthesis of GAcAc was performed in the region of maximum conversion at varying temperatures (from 70 to 80 °C) since the CCRD evaluation revealed a significant positive effect of this variable in the absence of 5Å MS. The enzyme amount (8.4%) and the substrate molar ratio (1:5) were kept constant, and the reaction was followed for 120 min. Figure 4a shows that all the reactions reached conversion values near the maximum after 60 min showing little significant increases after this time. The highest conversions measured after 60 min were 75, 80, 83, and 85%, at 70, 75, 80, and 85 °C, respectively. This kinetic study confirms the significant positive effect of temperature found in CCRD.

In addition, the experimental conversions measured after 30 min (73, 76, 80, and 81% at 70, 75, 80, and 85 °C, respectively) resulted very close to those calculated by using Eq. 1 (74, 76, 78 and 80% at the same temperatures) so, proving that the model furnished by the CCRD is predictive for the range of conditions studied.

To confirm that the temperature increase does not exert a positive effect on the conversion in reactions performed with 5Å MS, where the evaporation of methanol is not fundamental for its displacement from the reaction mixture, a kinetic evaluation was carried out under the same above conditions (enzyme amount 8.4%, substrates molar ratio 1:5, temperature from 70 to 80 °C) in the presence of 5Å MS. The results of this experiment are shown in Fig. 4b. The conversion values measured at different times did not change for reactions performed at different temperatures, with values greater than 90 and 95% achieved after 30 and 60 min, respectively.

Many studies have commented on the effect of methanol in lipase-catalyzed reactions. For example, Kulschewski et al. reported significant inhibition of free *Candida antarctica* lipase B for methanol concentrations in the reaction mixture greater than 1% [66]. On the other hand, Mangiagalli et al. demonstrated that the exposure of immobilized *Candida antarctica* lipase B (Novozym 435) to methanol concentration of up to 15% before the reaction did not result in activity loss [67]. These authors concluded that the methanol-induced lipase inactivation is due to a change in the protein conformation, which causes the aggregation of the enzyme and its detachment from the solid support; however, evidence of the influence of water activity, competitive inhibition, and effects on local conformational dynamics have been reported as well [66, 67]. The theoretical concentration of methanol in the reactions performed without 5Å MS, in the present study rage from 2 to 4% (w/w) for geraniol conversion values higher than 50%. Such concentrations are sufficient to inhibit the Lipo 435; thus, the positive effect of the temperature, which has been observed only in the absence of 5Å MS, can be attributed to faster removal of methanol by evaporation at higher temperatures.

### CCRD validation for microwave-assisted enzymatic synthesis of GAcAc with 5Å MS

**Fig. 5:**
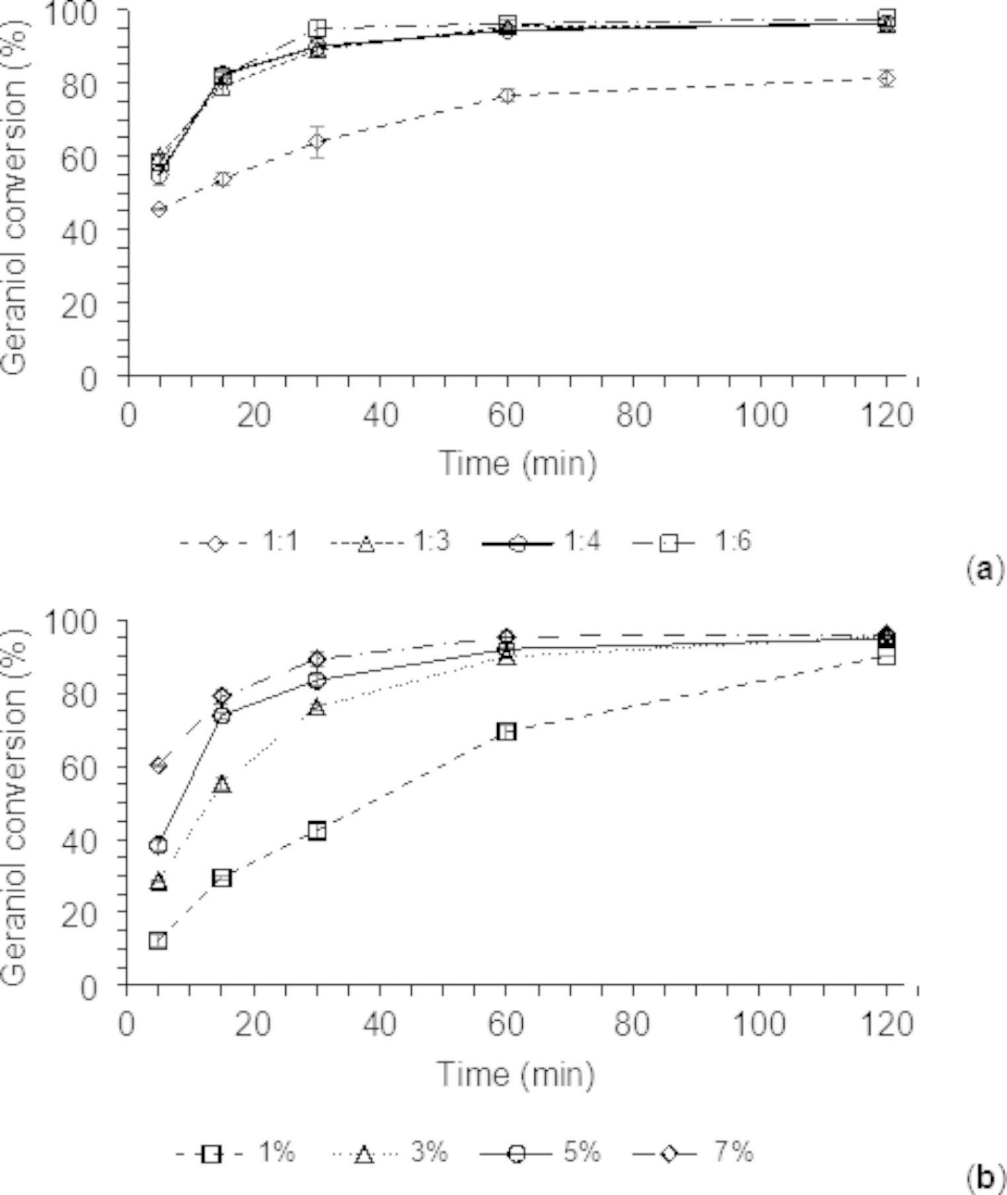
Kinetics of experimental validation conditions of molar ratio (**a**) and enzyme amounts (**b**) for microwave-assisted geraniol conversion with 5Å MS

Two kinetic studies were performed to validate the predictive model (Eq. 2) for the enzyme-catalyzed microwave-assisted GAcAc production in the presence of 5Å MS. The effects of different substrates’ molar ratios and enzyme amounts were evaluated by keeping the temperature constant (70 °C). Figure 5a shows the results obtained by changing the substrate’s molar ratio from 1:1 to 1:6. It can be noted that for values greater than 1:3, conversions above 95% were obtained after 60 min without further increases after this time. On the other hand, for the reaction carried out with a 1:1 molar ratio, the kinetic was slower, and the conversion values measured after 60 and 120 min were 76% and 81%, respectively (about 19 and 16% lower than those measured after the same times with the higher molar ratios).

The conversion values measured after 30 min with molar ratios greater than 1:3 was comprised between 90 and 95% and are very similar to those predicted by Eq. 2 (87, 89, and 92% for the molar ratios of 1:3, 1:4, and 1:6, respectively), confirming the good fit of the model with the experimental data. The difference between the conversion values achieved with 1:3 and 1:6 molar ratios was less than 6%, so the 1:3 condition was employed to validate the predictive model in the experiment with different enzyme amounts.

The biocatalysts cost is one of the primary limits for a broad application of lipases (and enzymes in general) in bioprocesses [65], so a kinetic study of the microwave-assisted enzymatic transesterification of methyl acetoacetate with geraniol in the presence of different amounts of the enzyme (1, 3, 5, and 7%) was carried out by keeping constant the substrates molar ratio (1:3) and the temperature (70 °C). It can be observed from Fig. 5b that for reaction times shorter than 60 min, increases in the biocatalyst amount determined significant increments of the conversion while, after 60 min, the three reactions performed with an enzyme amount ≥ 3% showed similar conversions values comprised between 89% and 95%.

The conversions predicted by the model after 30 min were 51, 72, 84, and 87%, with enzyme amounts of 1, 3, 5, and 7%, respectively. The experimentally obtained conversions were 42, 76, 83, and 89%, showing an excellent fit of the model to the experimental results.

The reaction using the enzyme amount of 1% is much slower; however, after 120 min, it reached a conversion of 90%, which is only 5% lower than the conversion value obtained after the same time with the higher enzyme amounts (approximately 95%). From these results, the selection of the enzyme amount can be performed in two ways; by using high enzyme amounts (≥ 5%) and short reaction times (≤ 60 min) or by using small enzyme amounts (≤ 1%) and longer reaction times (≥ 120 min). This choice must be considered in optimizing the process from an economic point of view.

### Lipo 435 reusability

**Fig. 6:**
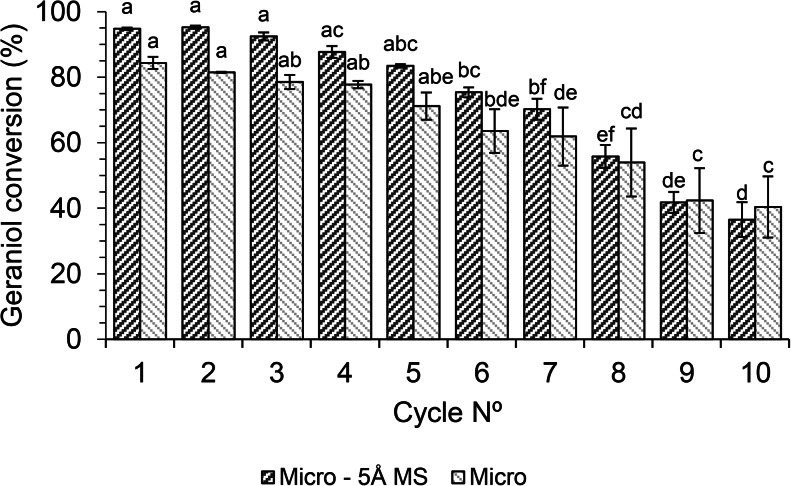
Biocatalyst reusability under microwave irradiation. Reaction conditions: 1:5 molar ratio, 80 °C, 8.4% of enzyme in the absence of 5Å MS (Micro), and 1:3 molar ratio, 70 °C, 7% of enzyme in the presence of 5Å MS (Micro – 5Å MS). Reaction time 60 min. The values with a different letter (**a–f**) in the bar of the same color are significantly different (p ≤ 0.05)

As stated above, the cost of the biocatalyst strongly affects the economic sustainability of biotechnological industrial processes. Therefore, a study on the biocatalyst reuse was carried out under the CCRD optimized conditions that for the system without 5Å MS were a substrate molar ratio of 1:3, 70 °C and 7% of the enzyme, while for the system with 5Å MS, were a molar ratio of 1:5, 80 °C and 8.4% enzyme. Figure 6 shows that the conversion values reached in the first 5 cycles were constant in both the reaction systems, as confirmed by the *t-test*. However, after the 5th reuse, a continuous decrease in the conversion was observed until the 10th cycle, with an overall loss of 55% and 50% in the reaction with and without 5Å MS, respectively. These results demonstrated the good stability of the Lipo 435 under microwave irradiation, which agrees with that previously reported for the Novozym 435 in the microwave-assisted synthesis of geranyl cinnamate, butyl acetoacetate, (*R,S*)-flurbiprofen and isoamyl myristate [57, 63, 68, 69]. The drop in the conversion after the 5th cycle may be linked to loss of activity due to prolonged exposure to substrates and product, thermal effects, loss of water from the enzyme microenvironment, the support pores’ blockage makes it difficult for reagents to access the active site, resistance to mass transfer and intraparticle diffusion, and the failure of support integrity disrupting the bond between the support and the enzyme [69–71]. Furthermore, the enzyme support destroying can be the result of the mill effect due to the less resistance to attrition of the biocatalyst acrylic resin with respect to 5Å MS.

### Microwave-assisted enzymatic synthesis of other geranyl esters

**Fig. 7:**
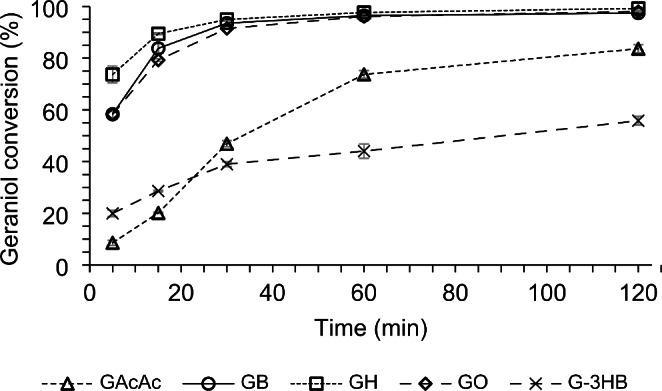
Microwave-assisted enzymatic synthesis of various geranyl esters. Reaction conditions: molar ratio of 1:3, 70 °C, 7% of enzyme amount, and 5Å MS. GAcAc: geranyl acetoacetate, GB: geranyl butyrate; GH: geranyl hexanoate; GO: geranyl octanoate; G-3HB: geranyl (*R*)-3-hydroxybutyrate

The microwave-assisted enzymatic transesterifications of ethyl acetoacetate, ethyl butyrate, methyl hexanoate, methyl octanoate, and methyl (*R*)-3-hydroxybutyrate with geraniol were performed under the optimized conditions for the synthesis of GAcAc with methyl acetoacetate in the presence of 5Å MS (Fig. 7). The production of GAcAc using the ethyl ester of the acetoacetic acid showed a much slower kinetic in comparison to the reaction carried out with the corresponding methyl ester (Fig. 5). Conversion values of 47 and 77% were obtained after 30 and 60 min, respectively, and a maximum of 83% was reached after 120 min. Another ketone body ester tested as the acylating agent was the methyl (*R*)-3-hydroxybutyrate. The synthesis of the corresponding geranyl (*R*)-3-hydroxybutyrate (G-3HB) showed a very slow kinetic, which reached a conversion of 30% after 30 min and a maximum conversion of 56% after 120 min. The reactions performed with the monofunctional linear esters ethyl butyrate, methyl hexanoate, and methyl octanoate reached higher conversions in shorter reaction times. The syntheses of geranyl butyrate (GB) and geranyl octanoate (GO) achieved conversion values greater than 58% after 5 min while, after the same time, a 73% conversion was obtained in the synthesis of geranyl hexanoate (GH) (Fig. 7). These three last reactions displayed a very similar kinetic behavior with conversion values of 93% after 30 min and greater than 96% and 98% after 60 and 120 min, respectively. Similar results (89%) have been reported by Chu et al. for the enzymatic microwave-assisted production of geranyl butyrate under continuous mode [62].

## Conclusions

The feasibility of microwave-assisted enzymatic synthesis of novel geraniol esters in a solvent-free system was successfully established in this study. The microwave-assisted transesterification of methyl acetoacetate with geraniol performed under optimized conditions reached 85% or 97% conversion values depending on the presence or absence of 5Å MS for removing the coproduced methanol. The enzymatic synthesis of GB, GH, and GO performed under the same conditions gave conversions greater than 98%, while the synthesis of G-3HB reached the maximum conversion of 56%. In addition, the lipase Lipo 435 displayed good operational stability under the optimized reaction conditions, remaining stable for 5 successive reaction cycles and maintaining approximately 40% of the original activity after 10 cycles. The results of the optimization, kinetics, and biocatalyst reuse studies presented in this work can provide insight into microwave-assisted biocatalytic processes furnishing a model approach for the implementation of bioprocesses devoted to the preparation of bioactive esters. From an economic and environmental point of view, the benefits of the process’s intensification provided by the combined use of microwave irradiation and enzymatic catalysis allowed the development of cleaner and safer eco-sustainable bioprocesses.

### Electronic supplementary material

Below is the link to the electronic supplementary material.

Supplementary Material 1
