# Supplementary material for: Microwave-assisted enzymatic synthesis of geraniol esters in solvent-free systems: optimization of the reaction parameters, purification and characterization of the products, and biocatalyst reuse
Source: Mol Divers. 2023 Jun 27;28(3):1665–79. doi: 10.1007/s11030-023-10682-y (PMC11269508; doi:10.1007/s11030-023-10682-y)
Supplement: Supplementary file 1 — Supplementary Material 1 [file 11030_2023_10682_MOESM1_ESM.docx]

*Supplementary Information*

**Microwave-assisted enzymatic synthesis of geraniol esters in solvent-free systems: Optimization of the reaction parameters, purification and characterization of the products, and biocatalyst reuse**

Valentina Venturi^1^, Francesco Presini^2^, Claudio Trapella^2^, Olga Bortolini^1^, Pier Paolo Giovannini^2^, Lindomar Alberto Lerin^2^*

^1^ Department of Environment and Prevention Sciences, University of Ferrara – UNIFE, Via Luigi Borsari, 46, 44121 – Ferrara – Italy

^2^ Department of Chemical, Pharmaceutical and Agricultural Sciences, University of Ferrara – UNIFE, Via Luigi Borsari, 46, 44121, Ferrara – Italy

*** Corresponding author**: Lindomar Alberto Lerin, lrnldm@unife.it

| 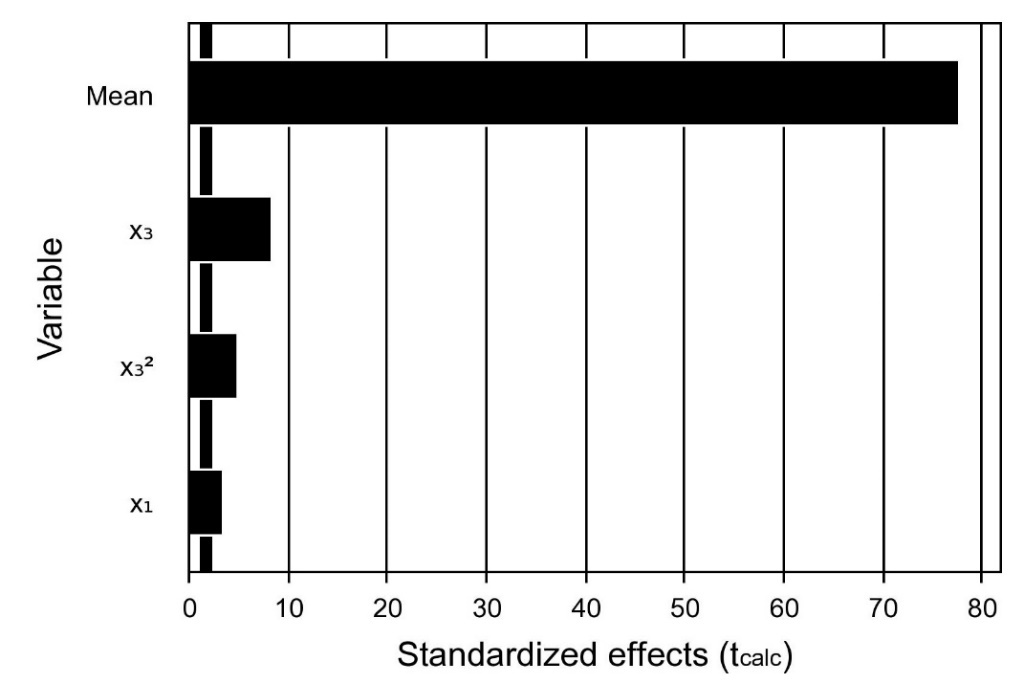 **(a)** |
| --- |
| 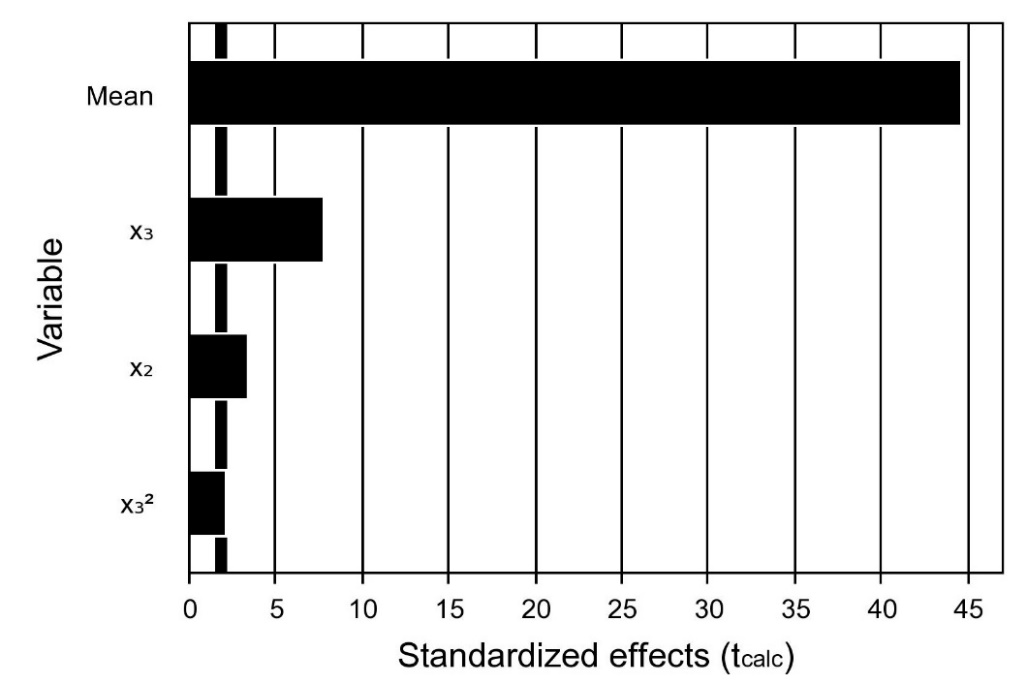 **(b)** |

**Figure S1** - Pareto chart with the significant effects of the independent variables studied on the microwave-assisted enzymatic synthesis of geranyl acetoacetate (GAcAc) in a solvent-free system with (a) and without (b) the removal of co-produced methanol (p<0.1). Where X_1_ is the molar ratio, X_2_ is temperature, and X_3_ is the enzyme amount. Experimental data and conditions are shown in Table 1.

| 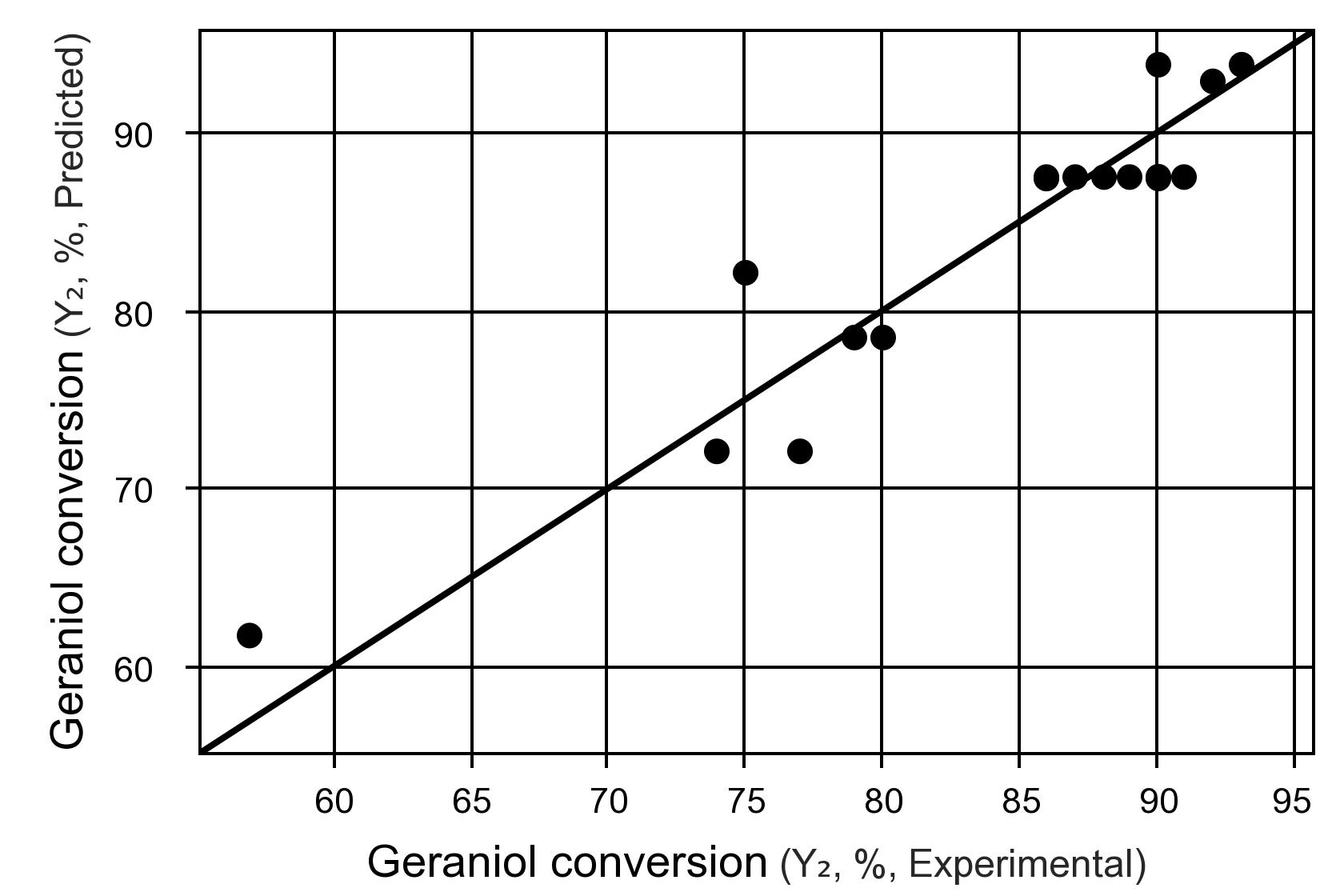 **(a)** |
| --- |
| 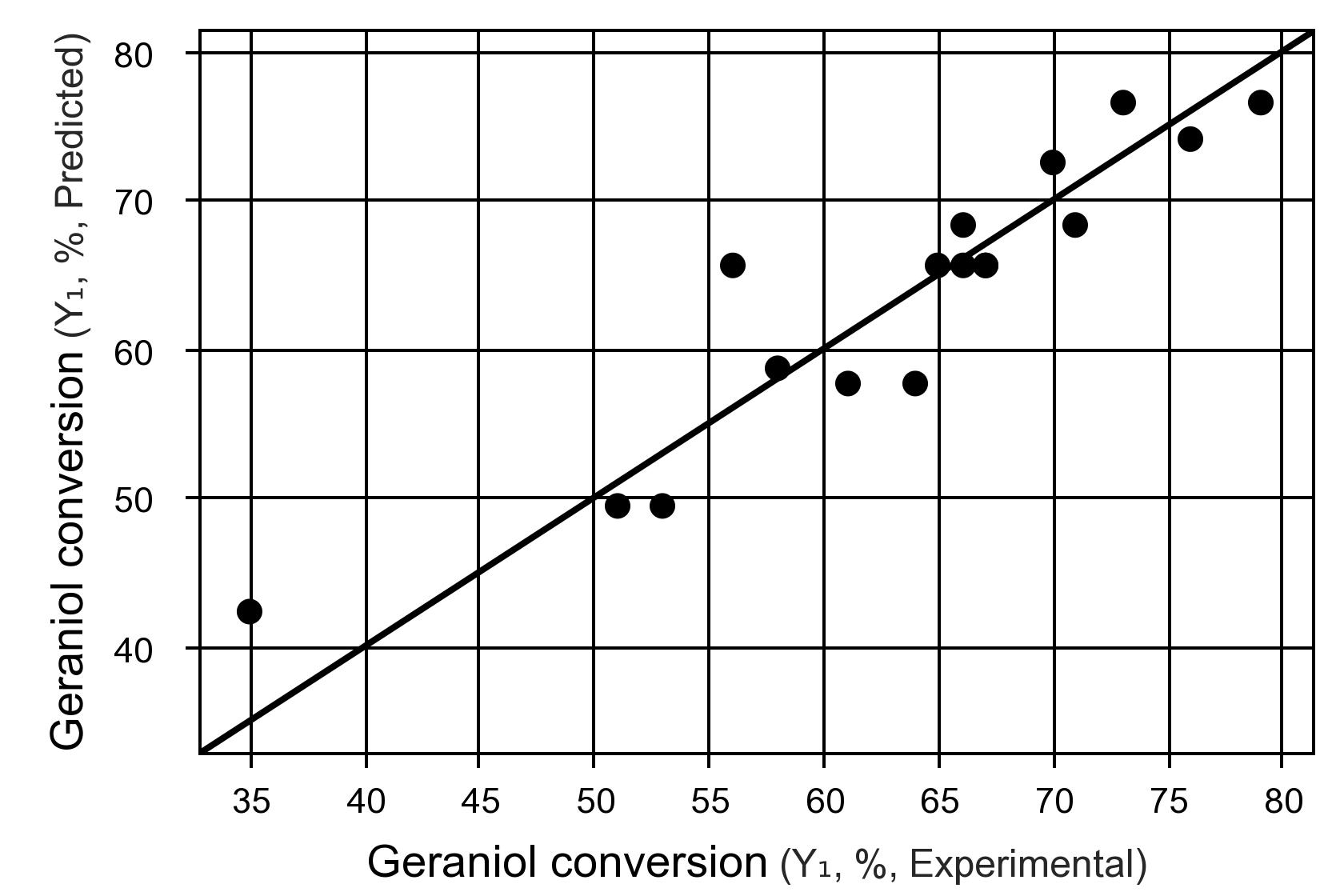 **(b)** |

**Figure S2** - Experimental versus predicted geraniol conversion for microwave-assisted enzymatic synthesis of GAcAc with (a) and without (b) removal of the co-produced methanol.

**Table S1** - Analysis of variance (ANOVA) of the estimated model for the optimization of microwave-assisted enzymatic synthesis of GAcAc with the removal of co-produced methanol.

| **Variation source** | **Sum of square** | **Degrees of**  **freedom** | **Mean square** | **F_calc_** | **P-value** |
| --- | --- | --- | --- | --- | --- |
| Regression | 1207.57 | 3 | 402.52 | 34.54 | 0.000001 |
| Residuals | 151.47 | 13 | 11.65 |  |  |
| Lack of Fit | 149.47 | 11 | 13.58 | 13.58 | 0.0704 |
| Pure Error | 2.00 | 2 | 1.00 |  |  |
| Total | 1359.05 | 16 |  |  |  |

Regression coefficient: R^2^ = 0.89; F_0.90; 3; 13_ = 2.56

**Table S2** - Analysis of variance (ANOVA) of the estimated model for optimizing microwave-assisted enzymatic synthesis of GAcAc without removing co-produced methanol.

| **Variation source** | **Sum of square** | **Degrees of**  **freedom** | **Mean square** | **F_calc_** | **P-value** |
| --- | --- | --- | --- | --- | --- |
| Regression | 1537.73 | 3 | 512.57 | 25.78 | 0.00001 |
| Residuals | 258.38 | 13 | 19.87 |  |  |
| Lack of Fit | 256.38 | 11 | 23.30 | 23.30 | 0.04183 |
| Pure Error | 2.00 | 2 | 1.00 |  |  |
| Total | 1796.11 | 16 |  |  |  |

Regression coefficient: R^2^ = 0.86; F_0.90; 3; 13_ = 2.56


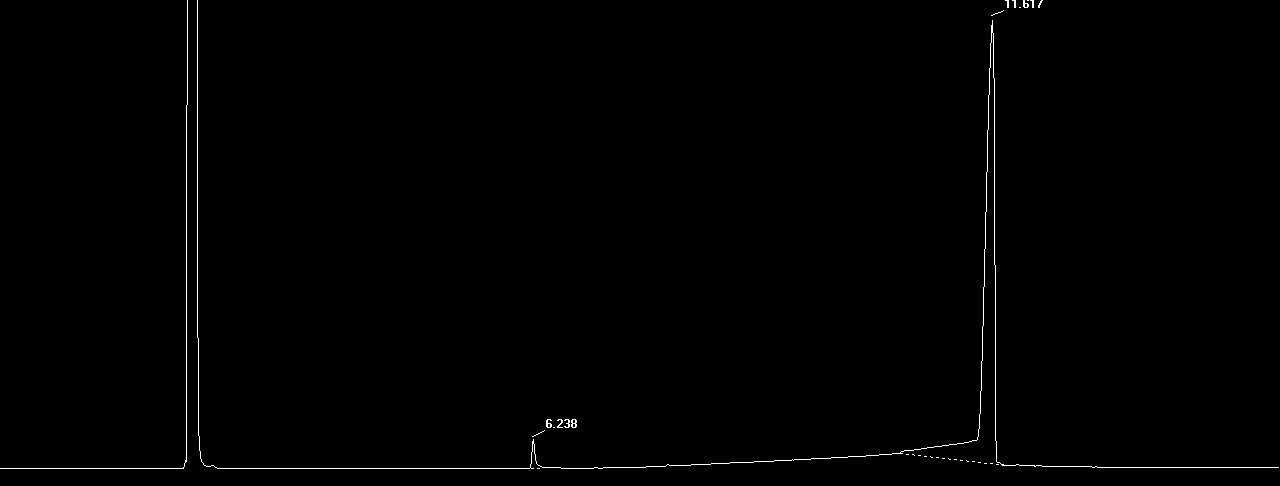


**Figure S3** - Typical gas-chromatogram of GAcAc after removal of the excess methyl acetoacetate by vacuum distillation (rotary evaporator, 70°C, 10 mbar). Retention time: ethyl acetate 2.94 min, geraniol 6.2 min and GAcAc 11.6 min.


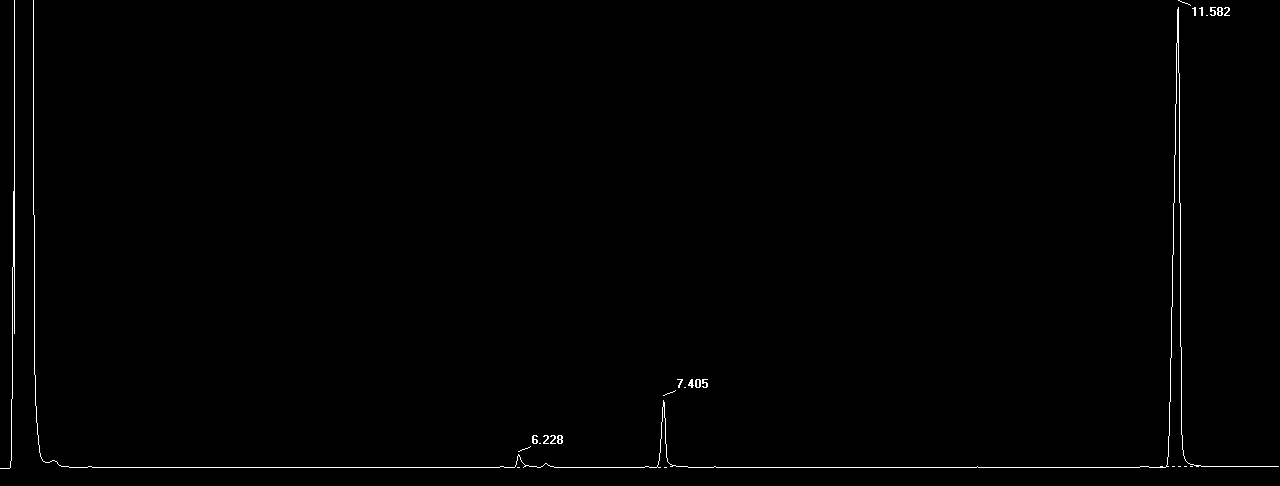


**Figure S4** - Typical gas-chromatogram of geranyl (*R*)-3-hydroxybutyrate (G-3HB) after removal of the excess of methyl (*R*)-3-hydroxybutyrate by vacuum distillation (rotary evaporator,70°C, 200 rmp, and 10 mbar). Retention time: ethyl acetate 2.94 min, geraniol 6.2 min, 4-methoxy-4-oxobutan-2-yl 3-hydroxy butanoate 7.4 min (see ^1^H-NMR spectra in Figure S13), and G-3HB 11.5 min.


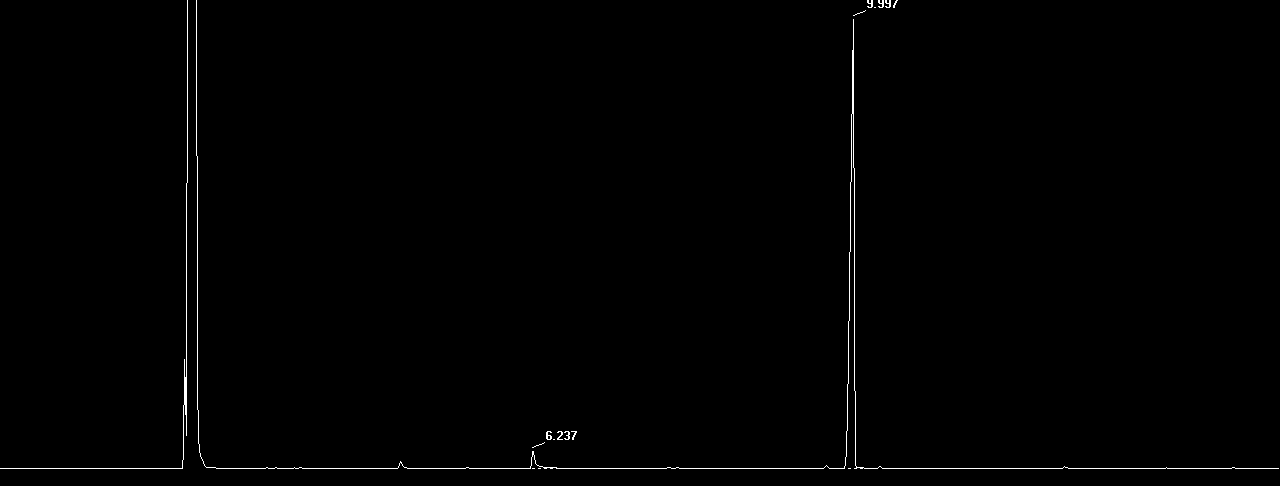


**Figure S5** - Typical gas-chromatogram of geranyl butyrate (GB) after removal of the excess ethyl butyrate by vacuum distillation (rotary evaporator, 70°C, 200 rmp, and 10 mbar). Retention time: ethyl acetate 2.94 min, geraniol 6.2 min, and GB 9.9 min.


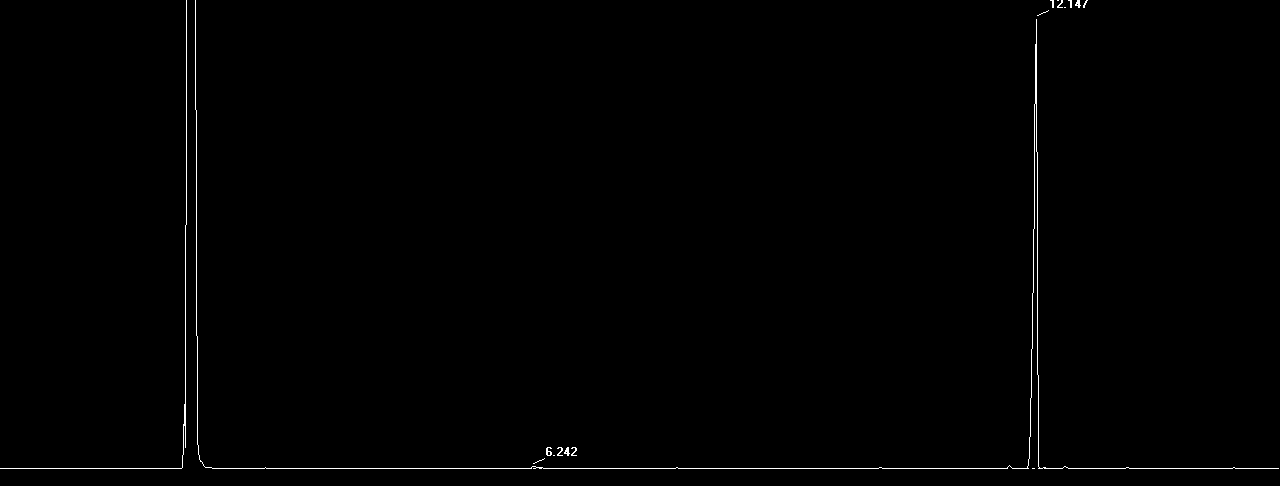


**Figure S6** - Typical gas-chromatogram of geranyl hexanoate (GH) after removal of the excess of methyl hexanoate by vacuum distillation (rotary evaporator, 70°C, 200 rmp, 10 mbar). Retention time: ethyl acetate 2.94 min, geraniol 6.2 min, and GH 12.1 min.


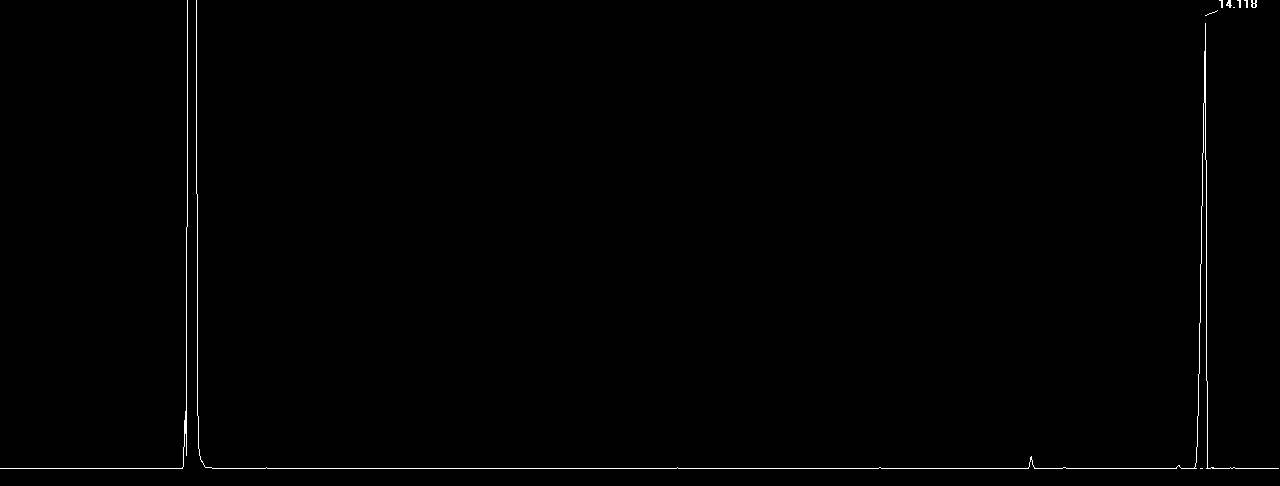


**Figure S7** - Typical gas-chromatogram of geranyl octanoate (GO) after removal of the excess of methyl octanoate by vacuum distillation (rotary evaporator, 70°C, 200 rmp, and 10 mbar vacuum). Retention time: ethyl acetate 2.94 min and GO 14.1 min.

| 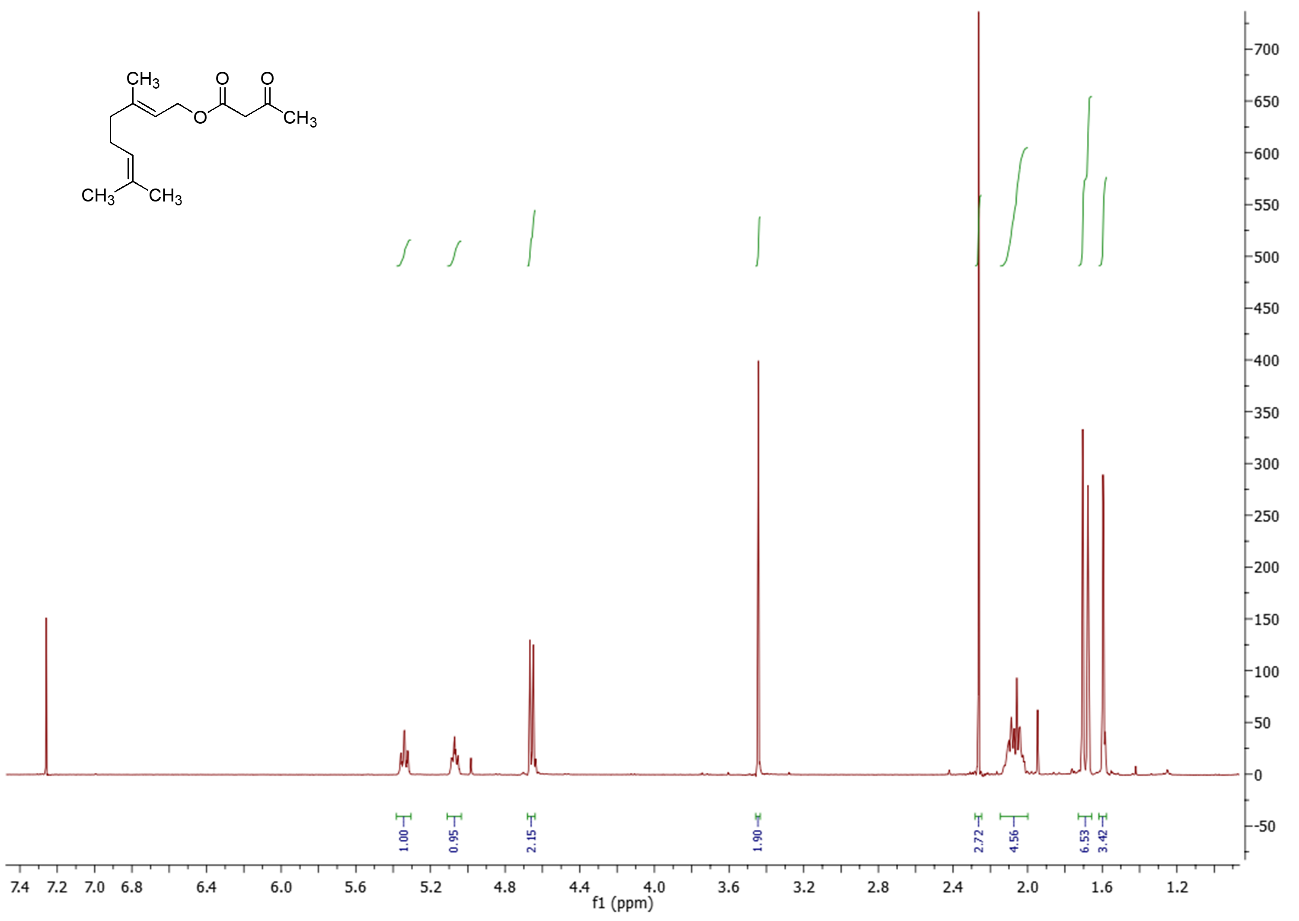 |
| --- |
| 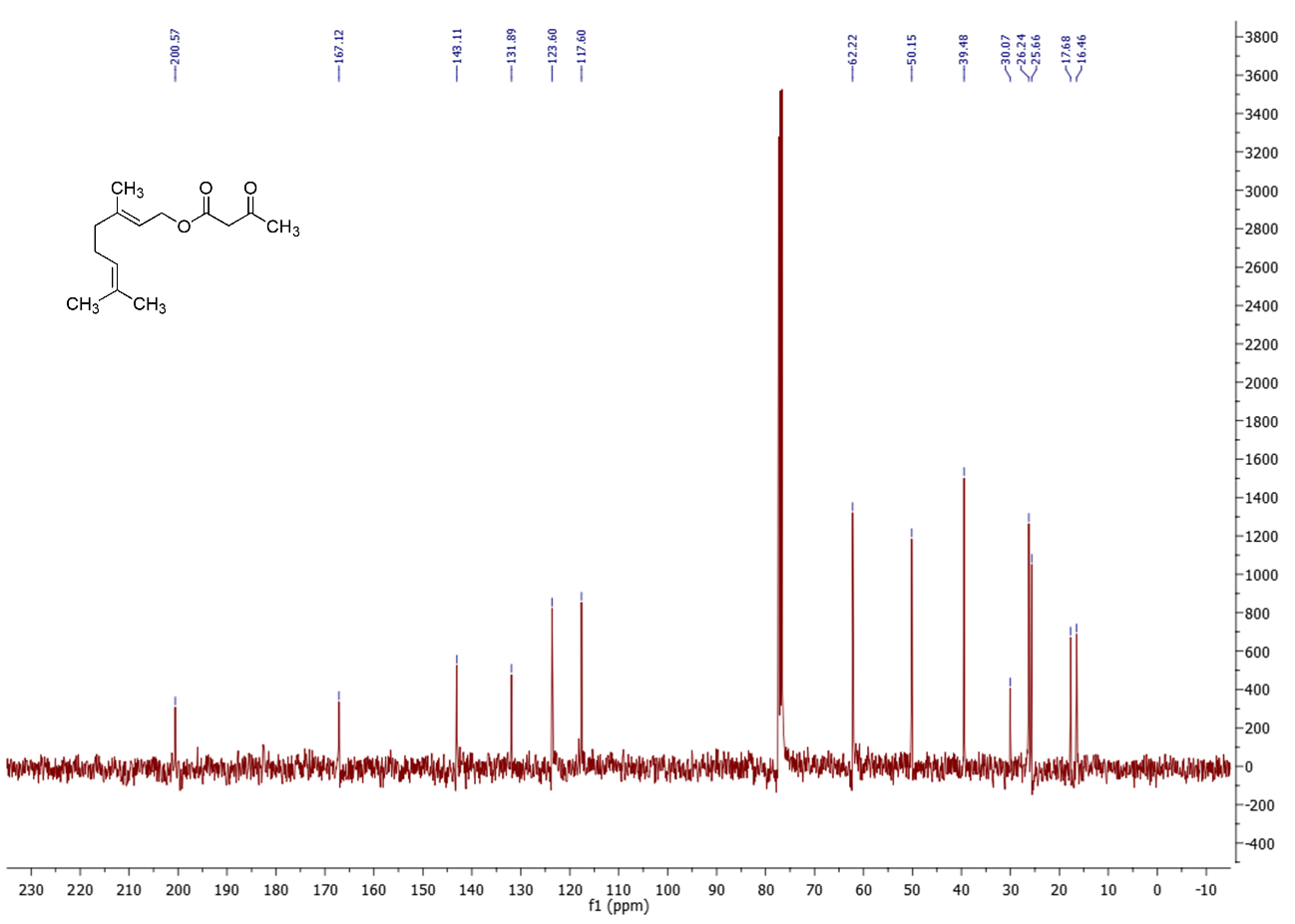 |

**Figure S8** - ^1^H- and ^13^C-NMR spectra of geranyl acetoacetate using CDCl_3_ at 400 MHz.

| 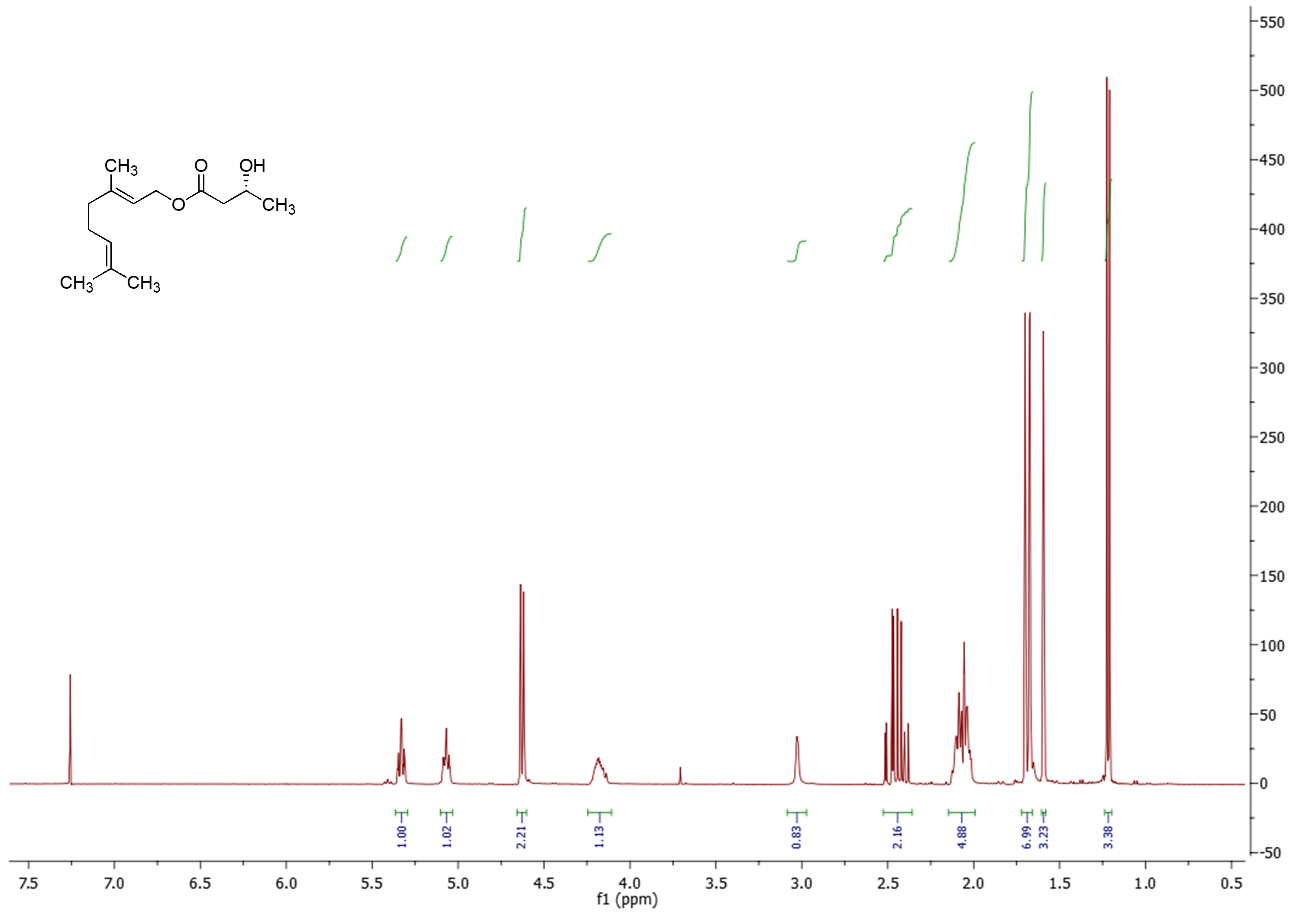 |
| --- |
| 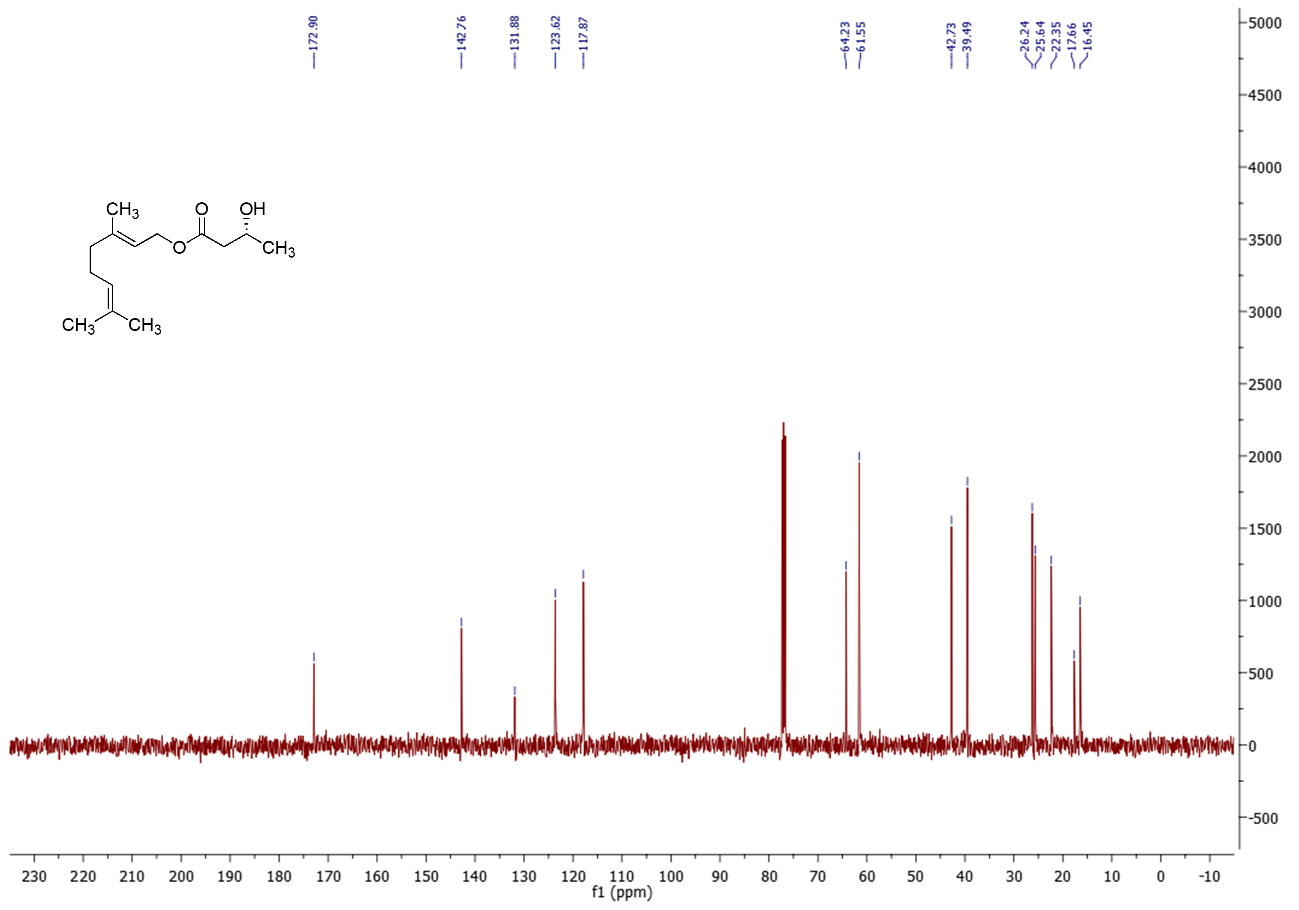 |

**Figure S9** - ^1^H- and ^13^C-NMR spectra of geranyl (*R*)-3-hydroxybutyrate using CDCl_3_ at 400 MHz.

| 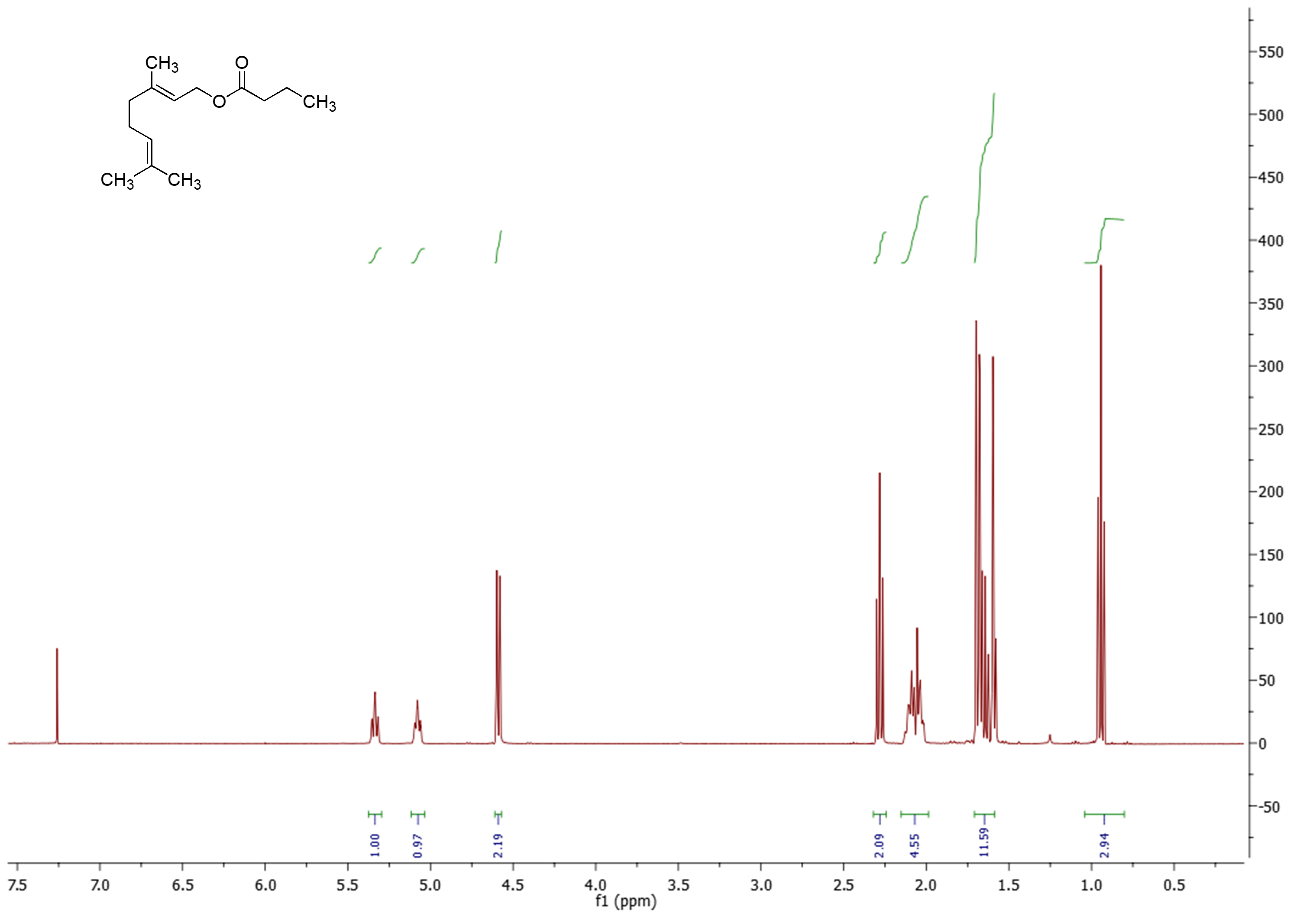 |
| --- |
| 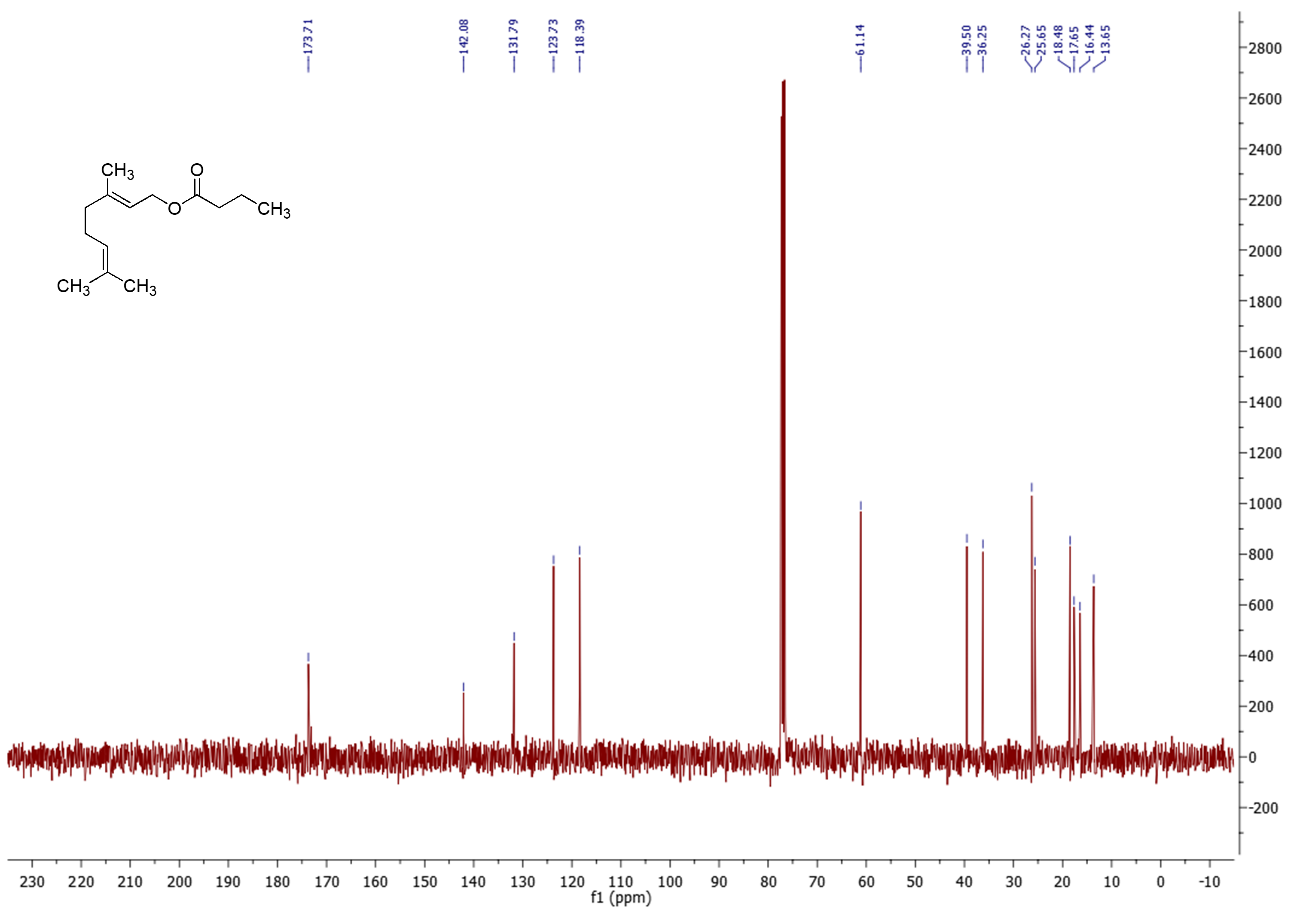 |

**Figure S10** - ^1^H- and ^13^C-NMR spectra of geranyl butyrate using CDCl_3_ at 400 MHz.

| 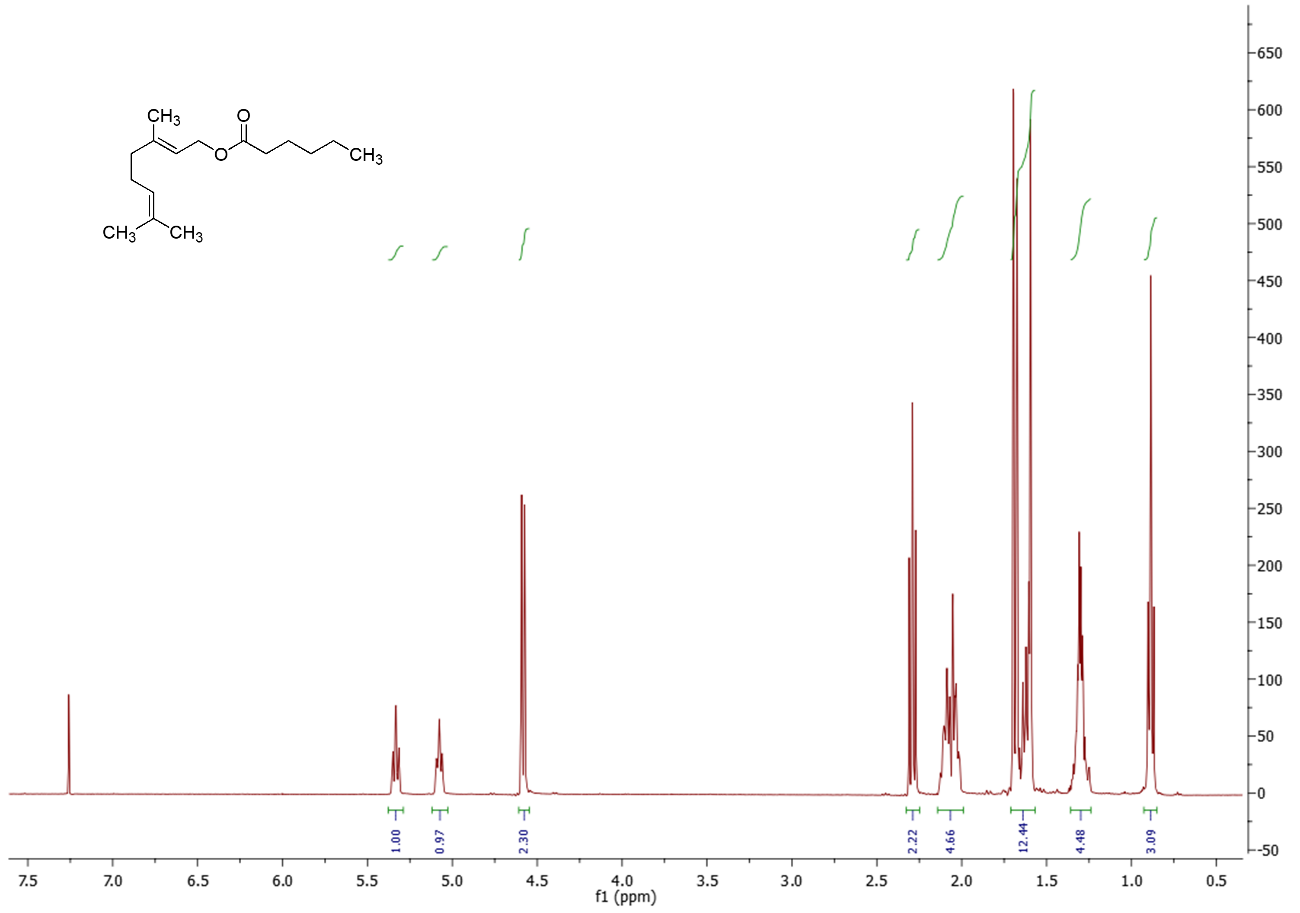 |
| --- |
| 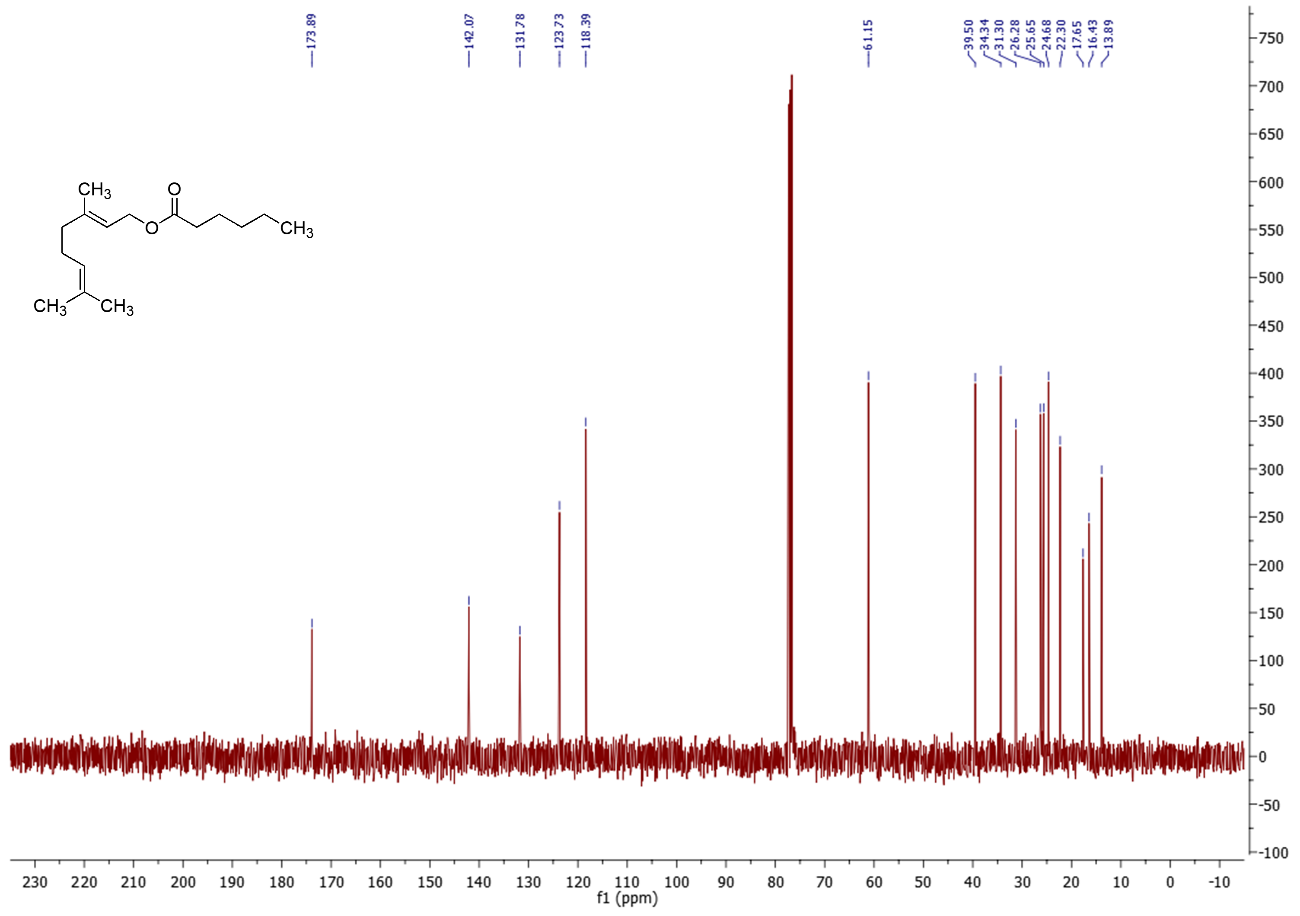 |

**Figure S11** - ^1^H- and ^13^C-NMR spectra of geranyl hexanoate using CDCl_3_ at 400 MHz.

| 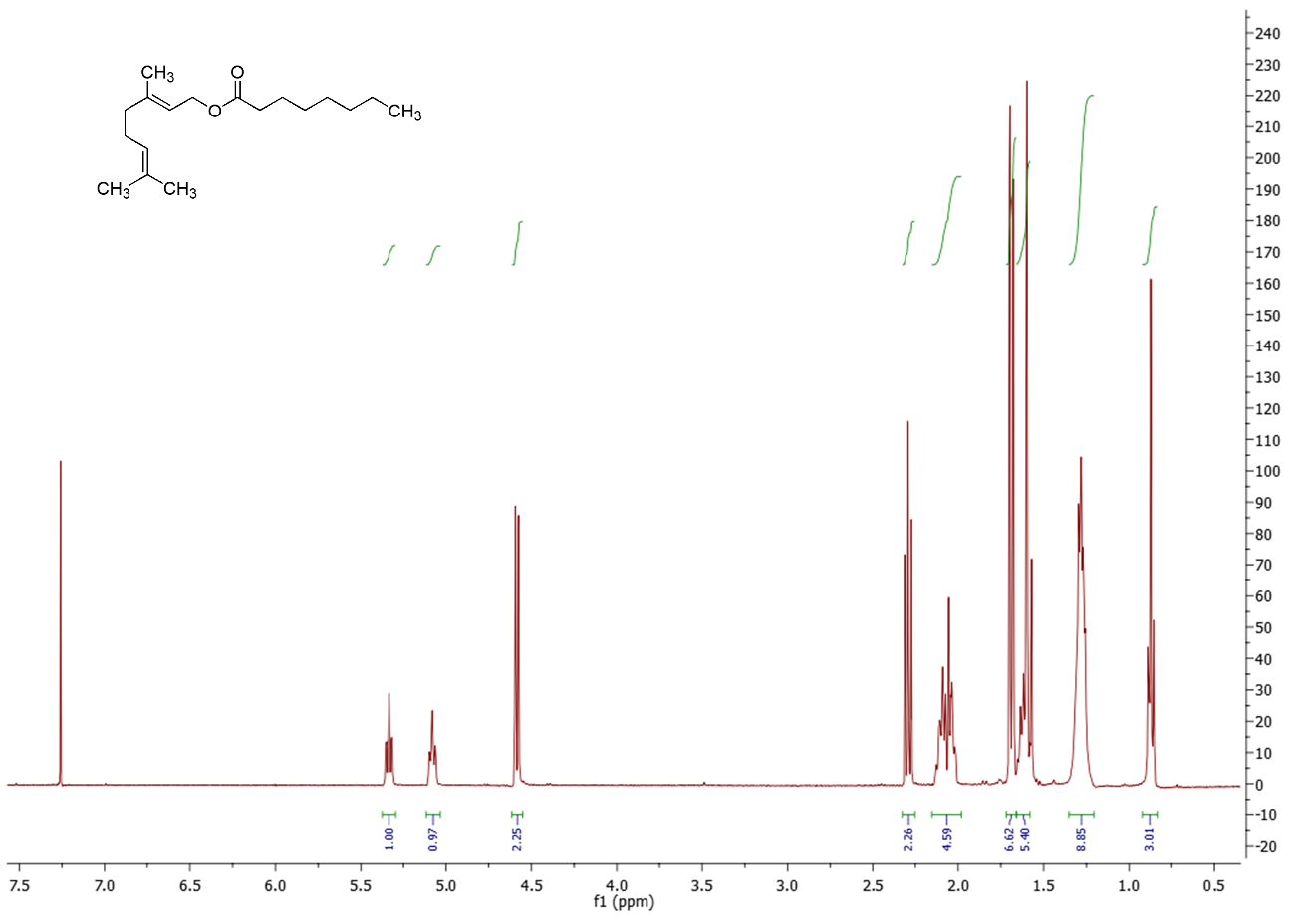 |
| --- |
| 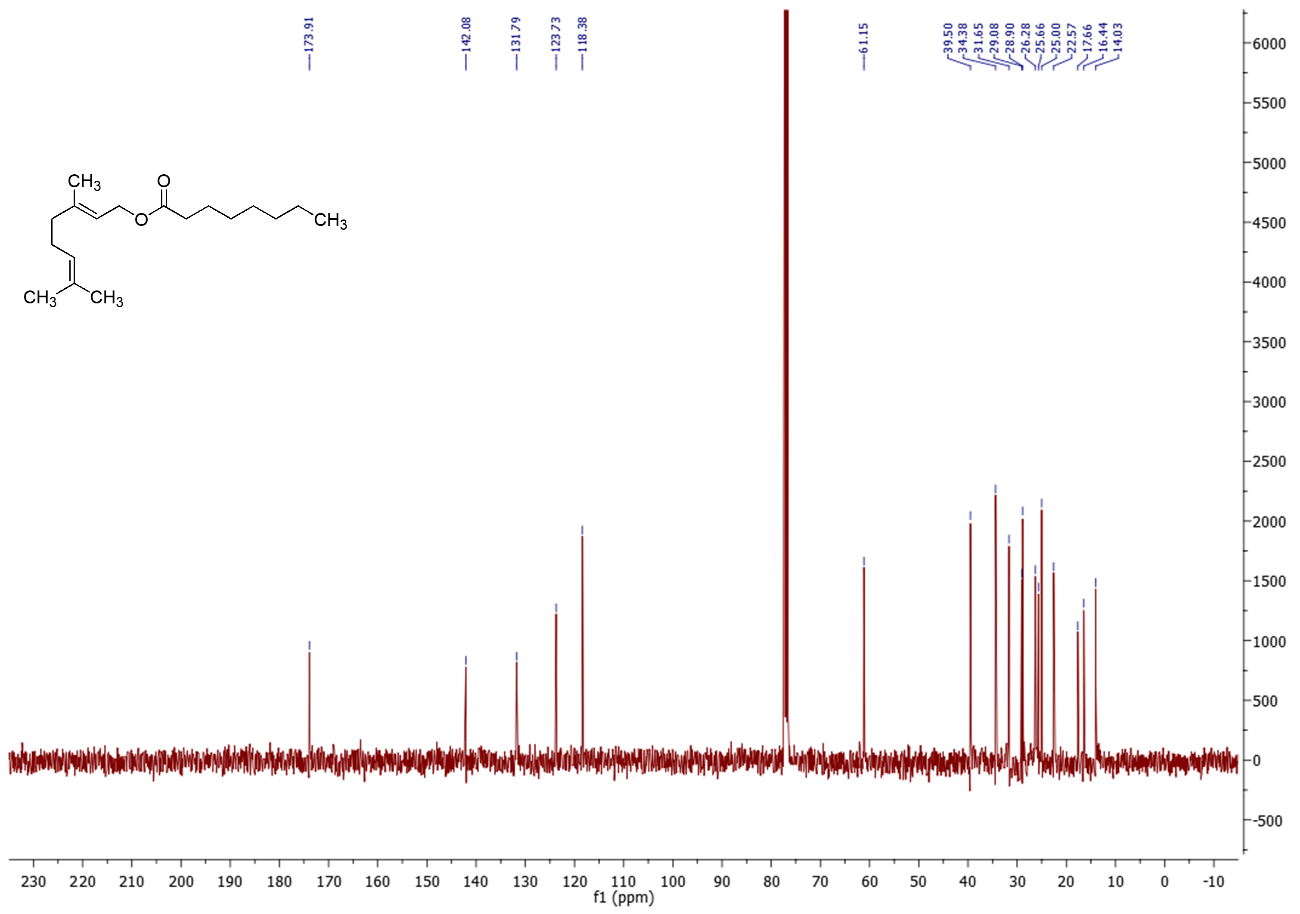 |

**Figure S12** - ^1^H- and ^13^C-NMR spectra of geranyl octanoate using CDCl_3_ at 400 MHz.


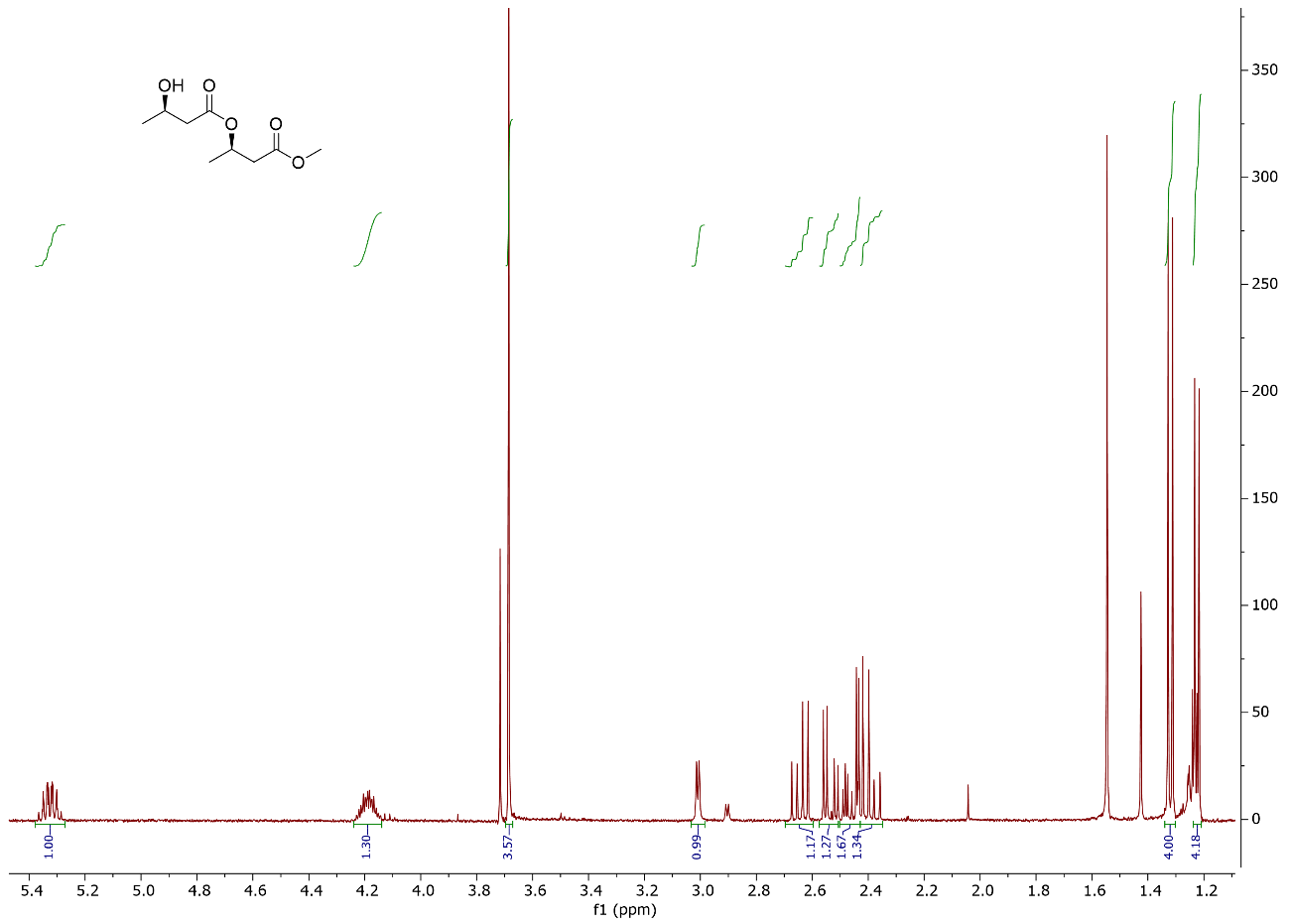


**Figure S13** - ^1^H-NMR spectra of 4-methoxy-4-oxobutan-2-yl 3-hydroxy butanoate using CDCl_3_ at 400 MHz: *δ* 5.37-5.28 (m, 1H), 4.23-4.14 (m, 1H), 3.69 (s, 3H), 3.01 (d, *J* = 3.6 Hz, 1H), 2.64 (dd, *J* = 15.6, 7.8 Hz, 1H), 2.53 (dd, *J* = 15.6, 5.3 Hz, 1H), 2.46 (dd, *J* = 15.9, 3.5 Hz, 1H), 2.39 (dd, *J* = 16.0, 8.5 Hz, 1H), 1.32 (d, *J* = 6.4 Hz, 3H), 1.22 (d, *J* = 6.3 Hz, 3H).
